# Dynamics of actinotrichia, fibrous collagen structures in zebrafish fin tissues, unveiled by novel fluorescent probes

**DOI:** 10.1093/pnasnexus/pgae266

**Published:** 2024-07-05

**Authors:** Junpei Kuroda, Hiromu Hino, Shigeru Kondo

**Affiliations:** Laboratory of Pattern Formation, Graduate School of Frontier Biosciences, Osaka University, Suita, Osaka 565-0871, Japan; Laboratory of Pattern Formation, Graduate School of Frontier Biosciences, Osaka University, Suita, Osaka 565-0871, Japan; Laboratory of Pattern Formation, Graduate School of Frontier Biosciences, Osaka University, Suita, Osaka 565-0871, Japan

**Keywords:** zebrafish, fin rays, collagen fibers, ECM, osteoclasts

## Abstract

Collagen fibers provide physical support to animal tissues by orienting in the correct position and at optimal density. Actinotrichia are thick collagen fibers that are present at the tips of fish fins and serve as scaffolds for bone formation. The arrangement and density of actinotrichia must be constantly maintained with a high degree of regularity to form spatial patterns in the fin bones, but the mechanisms of this process are largely unknown. To address this issue, we first identified two fluorescent probes that can stain actinotrichia clearly in vivo. Using these probes and time-lapse observation of actinotrichia synthesized at different growth stages, we revealed the following previously unknown dynamics of actinotrichia. (i) Actinotrichia do not stay stationary at the place where they are produced; instead, they move towards the dorsal area during the notochord bending and (ii) move towards the distal tip during the fin growth. (iii) Actinotrichia elongate asymmetrically as new collagen is added at the proximal side. (iv) Density is maintained by the insertion of new actinotrichia. (v) Actinotrichia are selectively degraded by osteoclasts. These findings suggest that the regular arrangement of actinotrichia is the outcome of multiple dynamic processes.

**Significance Statement**

The shape and function of animal tissues depend on the pattern of collagen fiber assembly. It is essential to clarify the dynamics of collagen fibers during tissue morphogenesis and growth, as abnormal size, density, and arrangement of collagen fibers can lead to severe dysfunction. However, this mechanism has not been elucidated due to the lack of suitable methods for analyzing collagen fiber dynamics in living tissues. Here, we describe a new method for live fluorescent labeling and time-lapse analysis of collagen fiber structures in zebrafish fins. Using this approach, we identified various novel dynamic behaviors of collagen fibers that were previously unknown. Our findings provide new insights into the study of collagen fiber in tissue morphogenesis.

## Introduction

Collagen, the most abundant protein in the animal body, plays a crucial role as a building material, particularly types I, II, and III fibrillar collagens, which are responsible for the physical properties of various tissues (1–3). In normal tissues, the quantity, thickness, and orientation of collagen fibers are strictly maintained by secreting collagen molecules in precise amounts, at precise locations, and timing. In the process of tissue morphogenesis, misassembly of collagen fibers causes morphological abnormalities (4–6). In addition, disruption of the balance between the fiber production and remodeling in various tissues such as the lungs, hearts, and kidneys cause fibrosis (4, 5, 7, 8). Furthermore, it is known that reorganization of collagen fibers plays a key role in cancer cell metastasis (9–11). Thus, to elucidate the mechanisms of tissue morphogenesis and disease, it is important to understand how fibrous structures of collagen are precisely constructed, and to achieve this, it is essential to clearly visualize them that are embedded within tissues.

Various methods have been used to observe collagen fibers in tissues, including histological staining, immune-antibody staining, and electron microscopy on fixed samples (12–18). Recently, second harmonic generation (SHG) imaging and GFP labeling techniques have enabled the observation of collagen fibers in vivo (19–26). However, there is still no useful method to easily and precisely observe the dynamics of collagen fibers in living tissues.

Actinotrichia, which are found in transparent fish fins, are of interest as rare experimental materials that allow easy observation of the dynamics of collagen fibers. They are collagen fiber structures with distinct spear-like shapes that are located at the tip of each fin ray (27, 28). Actinotrichia are thought to physically support the fin tissues by aligning regularly between epithelial and mesenchymal cells (29, 30). Moreover, since they are bundled at regular intervals slightly proximal to the tip of the fin, and bones are formed at these locations, they are speculated to determine the location of fin bone formation (31, 32). In addition, they also function as the scaffolds for the mesenchymal cell migration during early fin formation to generate proper pattern of fin bones (33–35). Therefore, actinotrichia play a central role in the formation and growth process of fins, and it is crucial to reveal how these collagen fibers are produced and arranged in the correct position and orientation to understand fin formation. Consequently, many research groups are conducting studies on actinotrichia.

Previous studies have successfully identified the various molecules that compose actinotrichia. Duran and colleagues found that the main components of zebrafish actinotrichia are type 1 collagen and type 2 collagen (36). Huang and colleagues identified type 9 collagen (Col9a1c), as the gene responsible for the *prp* mutation that causes actinotrichia hypoplasia (31). In fins of the transgenic (Tg) fish which specifically expressed GFP-fusion Col9a1c in mesenchymal cells, actinotrichia were fluorescently labeled, indicating that Col9a1c is also likely to be a component of actinotrichia (37). It is suggested that Col9a1c is required to align actinotrichia in the correct orientation since the orientation pattern of actinotrichia was abnormal in the *col9a1c* knockout line (31, 32). Molecules other than collagen have also been identified as components of actinotrichia. Akimenko's group identified the actinodin family genes that is involved with actinotrichia formation and named them *actinodin1* (*and1*) and *actinodin2* (*and2*) (38). Furthermore, it has been demonstrated by antibody staining and GFP labeling that And1 and And2 are also components of actinotrichia (30, 38–41).

As mentioned above, information regarding actinotrichia component factors has been accumulated. However, as with collagen in other organs, little is currently known about how those molecules play a role in the process of actinotrichia production at the proper position and the arrangement of actinotrichia in the correct orientation. Compared to other tissues and organs, zebrafish fins are transparent and have actinotrichia with shapes that are easy to see, which are extremely advantageous for observation. Therefore, if an appropriate imaging method can be developed, it should be possible to clarify the dynamics of actinotrichia during fin formation.

In this report, we introduce novel fluorescent probes that can very clearly label actinotrichia in living zebrafish fins in a simple manner. Our method has the following useful features: (i) actinotrichia can be fluorescently labeled simply by incubating fish in the diluted probe solution; (ii) probes are nontoxic and the fluorescence does not fade, making it possible to track the changes of actinotrichia position and morphology; (iii) it is possible to distinguish only actinotrichia present in a specific time frame by staining them with two-color probes. Using these probes, we attempted to observe the dynamics of actinotrichia and interactions with the cells involved in vivo. As a result, we discovered the following previously unknown dynamics of actinotrichia. (i) In the process of dorsal bending of the notochord, actinotrichia move dorsally and dynamically change the arrangement pattern. (ii) Actinotrichia continue to move toward the distal fin tip during fin growth. (iii) Actinotrichia exhibit an anisotropic growth pattern in which they elongate to the proximal side while moving toward the distal fin tip. (iv) The insertion of newly produced actinotrichia between old actinotrichia keeps the density of actinotrichia bundles constant. (v) Actinotrichia are selectively degraded by osteoclasts. This study is the first to specifically show the dynamic behavior of collagen fibers during tissue growth, and it will also provide new insights into the studies on the dynamics of collagen fibers in tissues other than fins.

## Results

### Two-color fluorescent probes, DAFFM and DAR4M, enabling high-resolution imaging of actinotrichia

**Fig. 1.**
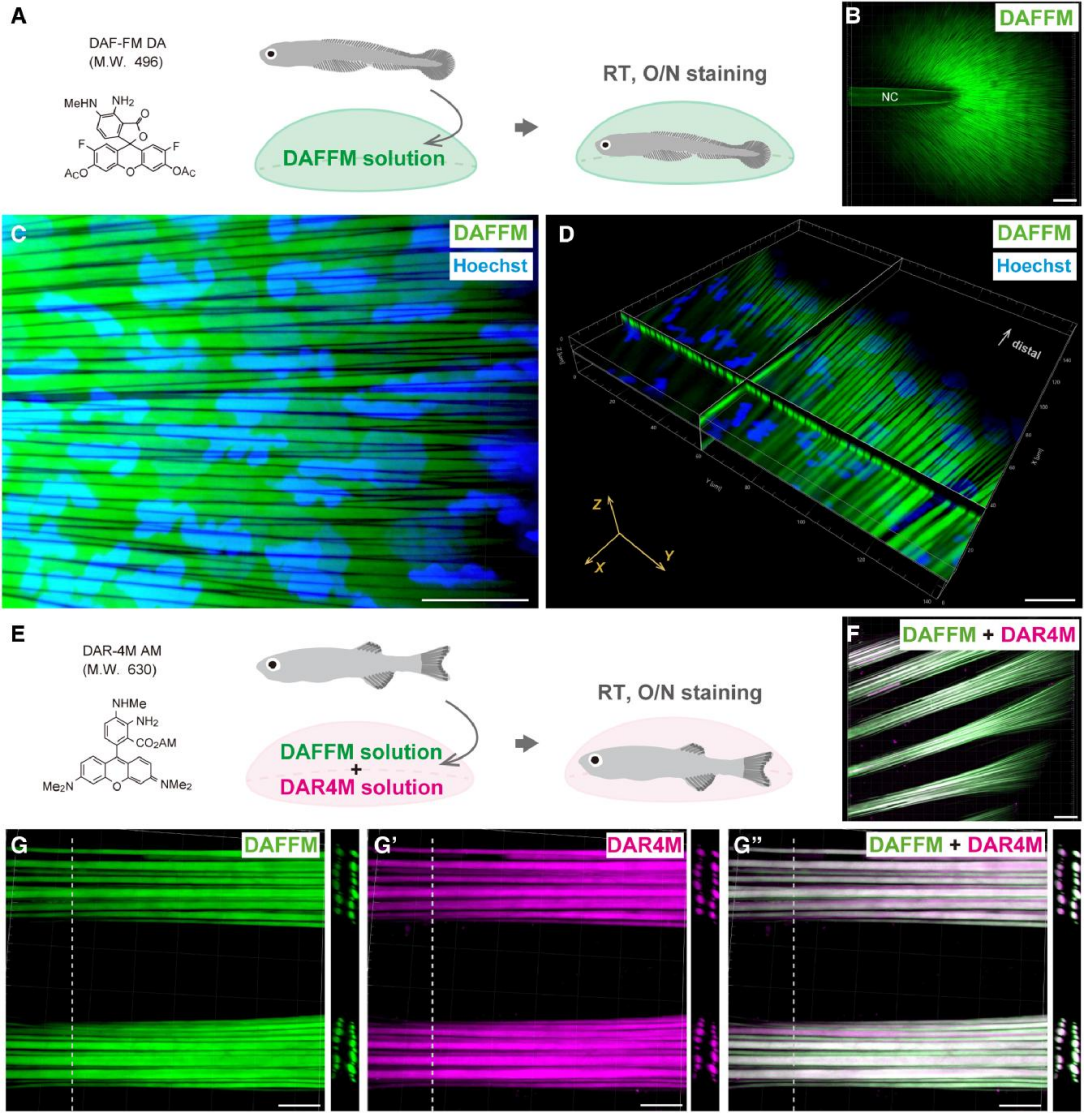
DAFFM/DAR4M enabling clear fluorescent labeling of actinotrichia with a simple method. (*A*) Schematic illustration of DAFFM staining for the actinotrichia visualization. Living larval zebrafish were incubated overnight in the DAFFM 5 μM solution at room temperature. (*B*) The fluorescent image of the larval caudal fin at 5 days post fertilization (dpf) stained with DAFFM. NC, notochord. (*C*) The magnified confocal image at center region of the caudal fin. Each actinotrichia was clearly visualized with DAFFM (green). Cell nuclei were stained with Hoechst (blue). (*D*) Cross-sectional views of the 3D reconstructed confocal image in the region from the center to the tip of the fin. (*E*) A schematic illustration of DAFFM and DAR4M staining for the actinotrichia visualization. Living young zebrafish were incubated overnight in the DAFFM 5 μM and the DAR4M 10 μM solution at room temperature. (*F*) The fluorescent image of the caudal fin tip at 21 dpf stained with DAFFM and DAR4M. (*G* to *G’’*) Magnified confocal images of the actinotrichia bundles at fin tip. The fluorescence of actinotrichia visualized by each probe is shown in (*G*) DAFFM (green), (*G’*) DAR4M (magenta), and (*G’’*) merged with DAFFM and DAR4M. Cross-sectional images at white dotted lines are shown on right panels. Scale bars, 50 μm (*B* and *F*) and 20 μm (*C*, *D,* and *G* to *G’’*).

Previous studies have shown that Diaminofluorescein-FM diacetate (DAF-FM DA) can be used to fluorescently stain cartilage tissues, bone and notochords in zebrafish (42–44). Since the actinotrichia of fins have not been evaluated in detail, we first investigated whether DAF-FM DA (hereafter referred to as DAFFM in this paper) can fluorescently label actinotrichia. To determine the optimal condition for visualization of actinotrichia using DAFFM, we tested various DAFFM staining conditions (SI Appendix, Supplementary Material text S1 and Fig. S1). As a result, the fluorescence intensity was highest when the fish were treated overnight (O/N, 12 hr) with 5 µM DAFFM solution (SI Appendix, Supplementary Material text S1 and Fig. S1), so we used O/N treatment for the fluorescent labeling of actinotrichia in this study (Fig. 1A). Under this staining conditions, strong fluorescence was observed in the notochord as in previous reports (42, 43), and whole-mount imaging using a confocal microscopy exhibited strong fluorescence in actinotrichia-like fibrous structures in the fins (Fig. 1B). The observation with high magnification revealed that the actinotrichia were clearly labeled such that each fiber can be individually distinguished (Fig. 1C and Movie S1). In the cross-sectional views of the confocal images, strong fluorescence was observed not only on the surface but also inside of the actinotrichia (Fig. 1D and Movie S1). Furthermore, we tested whether actinotrichia could be fluorescently labeled by DAFFM even when isolated from fin tissues. As a result, we found that they were indeed clearly labeled with DAFFM under in vitro condition (SI Appendix, Supplementary Material text S2 and Fig. S2).

Next, we examined whether the fluorescence of actinotrichia labeled with DAFFM accurately represented the shape of actinotrichia. Previous studies have reported that SHG imaging using multiphoton microscopy can be used for the visualization of actinotrichia in zebrafish larvae and allow the observation of their three-dimensional orientation within the fins (45–47). Therefore, we stained the actinotrichia in zebrafish larvae with DAFFM and compared the fluorescent signals of DAFFM with those of SHG (SI Appendix, Fig. S3). The fluorescence of DAFFM was observed in the same fiber structures that emit the SHG signal. However, although the SHG signal was nonuniform and partially undetectable on actinotrichia, DAFFM accurately and distinctly labeled the shape of actinotrichia (SI Appendix, Fig. S3).

Moreover, we evaluated whether Diaminorhodamin-4M acetoxymethyl ester (DAR-4M AM), a derivative of DAFFM, is similarly useful for fluorescent labeling of actinotrichia. To determine the optimal condition for visualization of actinotrichia using DAR-4M AM (hereinafter referred to as DAR4M in this paper), we tested various DAR4M staining conditions (SI Appendix, Supplementary Material text S1 and Fig. S4). As a result, actinotrichia emitted the strongest fluorescence under the conditions of the incubation O/N (12 hr) in 10μM solution (SI Appendix, Supplementary Material text S1 and Fig. S4). Therefore, using optimal conditions that allow for clear fluorescent staining of actinotrichia, we investigated whether DAFFM and DAR4M staining can be performed at the same time (Fig. 1E). When 3 weeks young fish were incubated in a solution of two fluorescent probes, the contours of actinotrichia were clearly marked with two fluorescent colors (Fig. 1F and G”). In addition, when observing the cross-sectional view, strong fluorescence was observed not only on the surface of actinotrichia but also inside, and the two fluorescence colors almost completely merged (Fig. 1G-G” and Movie S2).

We further tried to validate the utility of these probes for in vivo imaging. DAFFM and DAR4M have different fluorescence wavelengths (DAFFM: excitation 500 nm/emission 515 nm, DAR4M: excitation 550 nm/emission 572 nm) (48, 49). We then tested whether the fluorescence of actinotrichia labeled with these probes can be differentiated from the visualized cells expressing fluorescent proteins in the fins when simultaneously observed. As a result, we found that the fluorescent intensity of DAFFM-labeled actinotrichia and the cells expressing fluorescent proteins exhibited distinct peaks, allowing for their simultaneous observation (SI Appendix, Fig. S5). Based on these results, we concluded that DAFFM and DAR4M are powerful in vivo imaging tools that can easily fluorescently label actinotrichia, collagen fibers located inside fin tissues.

### Adaptation of DAFFM staining to live zebrafish tissues

We found that it is possible to observe the morphology and orientation of actinotrichia in high resolution and three-dimensionally using two fluorescent probes. To understand the mechanism by which the precise morphology of the fin is created, it is necessary to further analyze the dynamic changes in the distribution and morphology of actinotrichia using living fin tissues. Ideally, this should be accomplished by transiently labeling actinotrichia in living fish and using a pulse-chase observation method to observe the dynamics of labeled fibers over time. In addition, to perform this experiment, all of the following conditions must be achieved. (i) Staining treatment does not inhibit fin growth. (ii) Fluorescence does not fade during the growth process. (iii) Fluorescence does not diffuse from the fluorescently labeled area. Therefore, to investigate whether it is possible to use DAFFM to perform a pulse-chase observation of fluorescently labeled actinotrichia, we first investigated these three conditions.

**Fig. 2.**
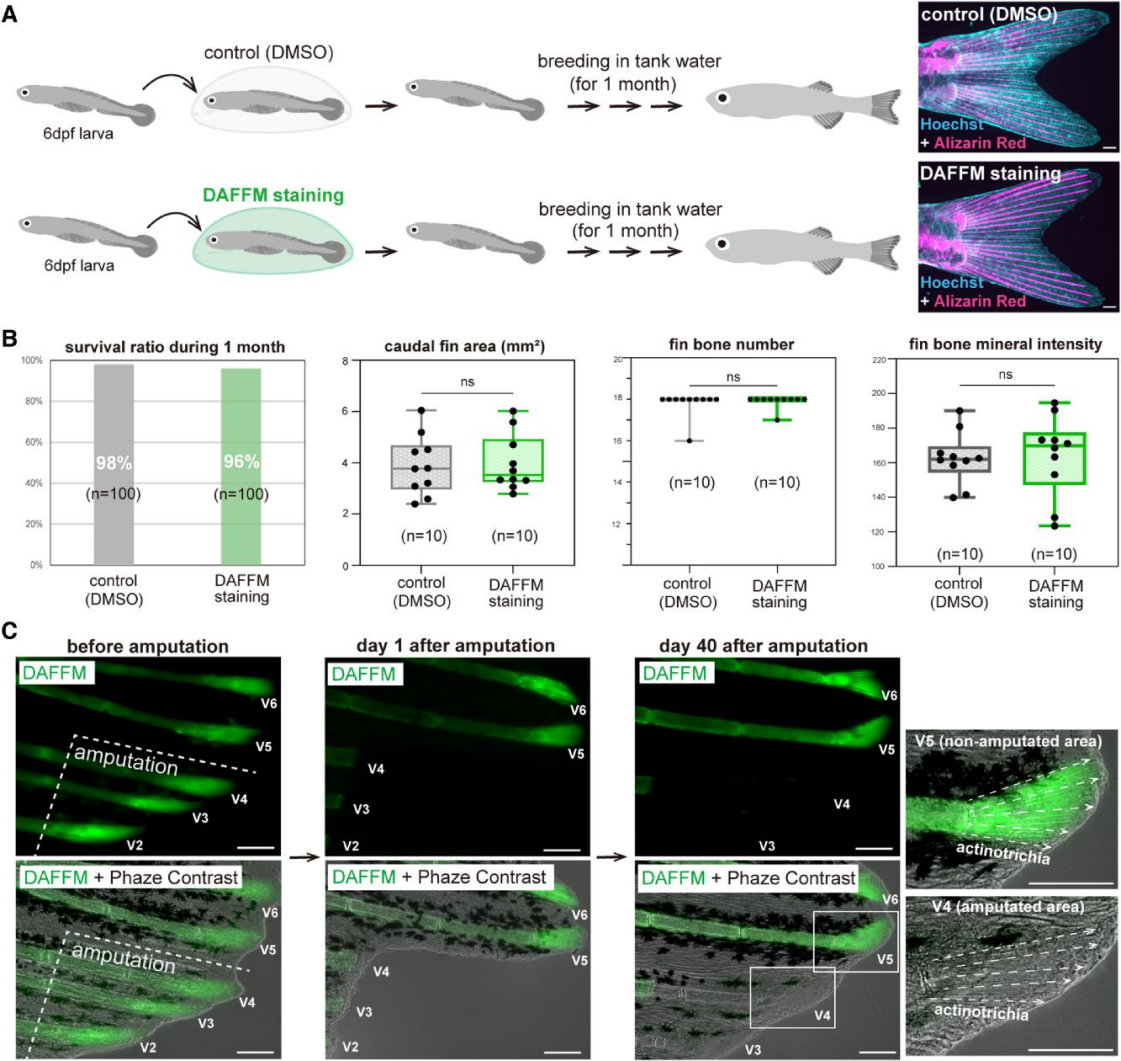
Powerful advantages of DAFFM staining for live observation of actinotrichia. (*A*) Schematic illustration of zebrafish treatments assessing the effect of DAFFM on fin development. After control (DMSO) treatment or DAFFM staining, the fish in both groups were returned to tank water and bred for one month. Subsequently, their fins were stained with Hoechst and Alizarin Red. (*B*) Comparison of survival ratio and fin growth between Control (DMSO) and DAFFM staining. (*C*) Mature adult fish (1.5-year-old) were treated with DAFFM and their actinotrichia were fluorescently labeled. After DAFFM staining, the fin tip region at the ventral side were amputated. They were bred for 40 days after fin amputation, and the fluorescence of actinotrichia at the tip of fins were observed. Data are means ± SD (unpaired Student's *t* test). ns indicates not significant. Scale bars, 200 μm (*A*) and 100 μm (*C*).

First, we investigated (i) whether the staining treatment does not inhibit fin growth. We divided 6 days post fertilization (dpf) larvae into two groups: no DAFFM staining (control: DMSO treatment) and DAFFM staining and returned them to breeding water after each treatment. They were grown for one month in breeding water and then compared for survival, fin size, number of fin bones, and fin bone mineralization (Fig. 2A and B). As a result, we found no significant difference in these measurements (Fig. 2A and B). In addition, we confirmed that any adverse effects on the activities of fin cells were not observed after DAFFM staining (SI Appendix, Fig. S6). These results suggest that transient fluorescent staining of living fish with DAFFM does not affect the fin tissue growth. Next, by conducting experiments on fin regeneration, we examined two conditions: (ii) whether fluorescence does not fade during the growth process, and (iii) whether fluorescence does not diffuse out from the fluorescently labeled area. We stained living adult fish with DAFFM, and amputated three fin rays (V2, V3, V4) after staining (Fig. 2C). We then returned them to breeding water and examined the fluorescence of the regenerating fin 40 days later. Surprisingly, we still observed strong fluorescence of DAFFM in the actinotrichia of the fin tip in the uncut region (Fig. 2C). Furthermore, we detected no fluorescence in the newly formed actinotrichia at the tips of the fins after regeneration (Fig. 2C). These results show that DAFFM staining achieves all three of the above conditions and is useful for understanding the dynamics of actinotrichia during fin growth.

### Pulse-chase observation using DAFFM to analyze the dynamics of actinotrichia during the growth process of living fish

**Fig. 3.**
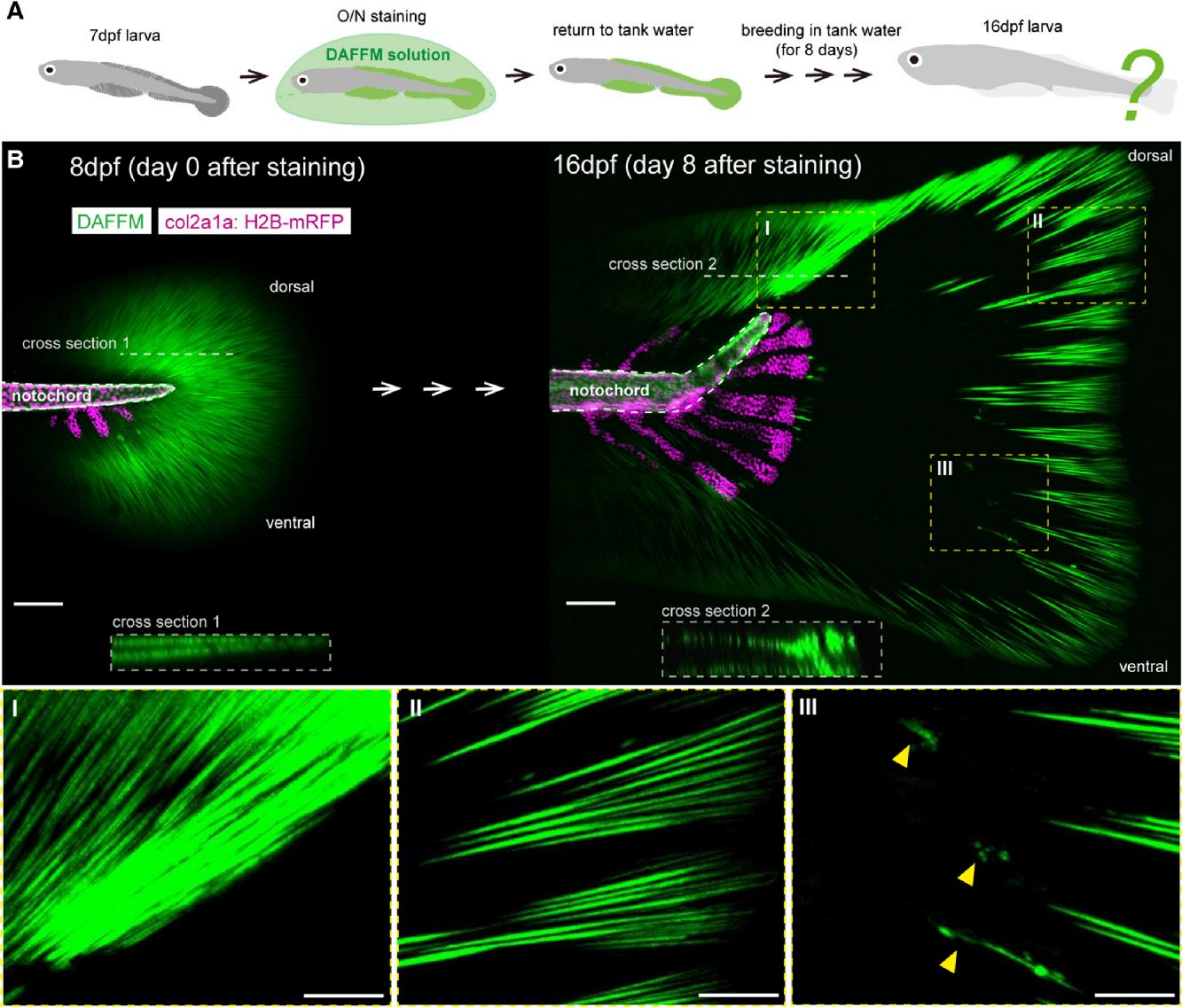
Pulse-chase observation of actinotrichia using DAFFM staining. (*A*) Workflow for the pulse-chase observation of actinotrichia by DAFFM staining during the early fin formation. Living 7 dpf larvae were incubated overnight in DAFFM solution and then returned to tank water. After breeding in tank water for 8 days, the fluorescence of actinotrichia in the caudal fin of the same fish were examined. (*B*) The fluorescence of actinotrichia labeled with DAFFM in the zebrafish fins at 8 dpf (day 0 after staining) and 16 dpf (day 8 after staining). The cell nuclei of notochord sheath cells and chondrocytes were visualized by the H2B-mRFP expression. Cross-sectional images at white dotted lines 1 and 2 are shown on lower panels. Yellow dotted boxes I, II, and III indicate actinotrichia bundled in the dorsal region, actinotrichia localized at the distal tip, and actinotrichia with irregular shapes, respectively. Yellow arrowheads indicate the actinotrichia with irregular shapes. Scale bars, 100 μm (*B*) and 50 μm (*B* I, II, and III).

We then attempted to do the pulse-chase observation of actinotrichia using DAFFM. We incubated living zebrafish larvae at 7 dpf in DAFFM solution and reacted them O/N. We then returned these DAFFM-labeled fish to tank water and bred them for 8 days (Fig. 3A).

Initially, we found that fluorescently labeled actinotrichia were arranged radially throughout the round-shaped tail fins at 8 dpf (Fig. 3B). In the process of early fin growth, the fins increased in size while changing shape, and surprisingly, their distribution pattern changed dramatically (Fig. 3B). At 8 dpf, actinotrichia formed two layers just below the basal epithelial cells of the fins (Fig. 3B, cross-section 1). However, at 16 dpf, we found that actinotrichia accumulated in bundles near the ends of the bent notochords in the dorsal region of the fins (Fig. 3B, dotted box I, cross-section 2).

During fin growth, the fins actively elongated toward the distal side, and notably, old actinotrichia labeled 8 days ago were located at the tips of the grown fins at 16 dpf (Fig. 3B, dotted box II). We also found that at 16 dpf, actinotrichia with markedly altered shape appeared in several regions (Fig. 3B, dotted box III). These remarkable changes in the distribution and shape of actinotrichia have not been previously reported. Therefore, we decided to engage a detailed study of these processes as shown in the dotted box I–III in Fig. 3B. We expected that this would provide insight into the dynamics of actinotrichia that support fin morphogenesis.

### Dynamic rearrangement of actinotrichia during notochord bending

**Fig. 4.**
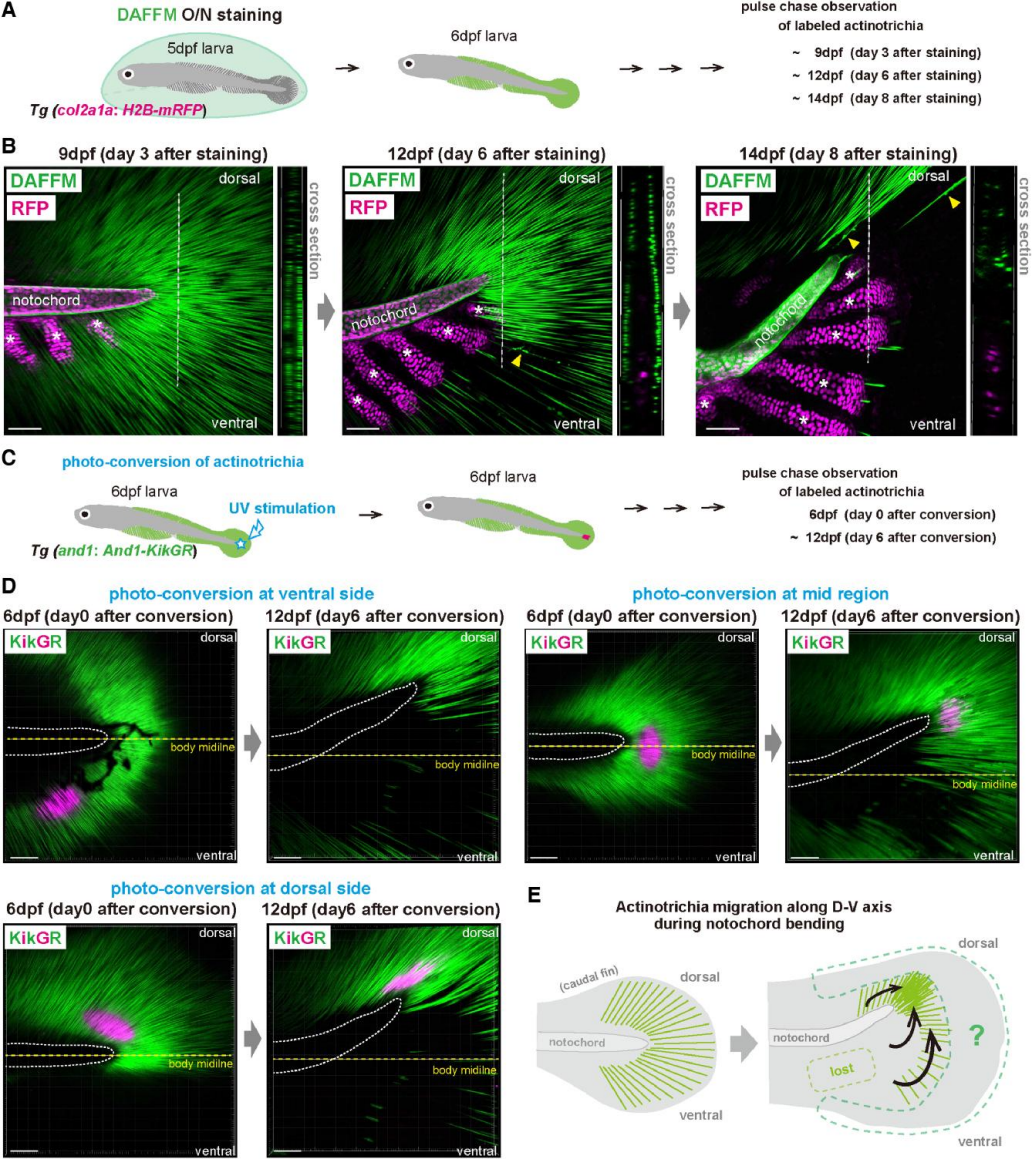
Actinotrichia dynamics during notochord bending. (*A*) Workflow for the pulse-chase observation of actinotrichia using DAFFM staining. (*B*) Confocal images of the fluorescently labeled actinotrichia in the caudal fin. Actinotrichia stained with DAFFM at 6 dpf were clearly visualized with green fluorescence. Nuclei of notochord sheath cells and chondrocytes were visualized by H2B-mRFP expression. Cross-sectional views at white dotted lines are shown on the right panel of each z-stacked image. Yellow arrowheads indicate the actinotrichia with irregular shapes. White asterisks indicate the primordia of cartilaginous tissues that develop at the base of caudal fins. (*C*) Workflow for the pulse-chase observation of actinotrichia using a photo-conversion system. (*D*) Confocal fluorescence images of actinotrichia after the photo-conversion. The fluorescent colors of actinotrichia in 6 dpf larval fins were changed by the photo-conversion at ventral side, mid region, and at dorsal side. Yellow dotted lines indicate the midline in the larval bodies. White dotted lines indicate the outline of notochords. (*E*) Summary diagram of the actinotrichia migration along D-V axis during notochord bending. Scale bars, 50 μm.

First, we investigated the changes in the distribution pattern of actinotrichia in the dorsal region during the pre and post-notochord bending stages by observing the same fish over time (Fig. 4A). To show the precise shape of the notochord, we used Tg (*col2a1a:H2B-mRFP*) fish that visualize the nuclei of notochord sheath cells and chondrocytes for DAFFM labeling (Fig. 4B). At stage 9 dpf, before notochords bend dorsally, actinotrichia are regularly oriented in a planar manner throughout the fins across the dorsal and ventral regions (Fig. 4B). However, when the notochord bending started, actinotrichia accumulated around the edge of notochords in the dorsal region (Fig. 4B). In addition, cross-sectional images showed that the arrangement pattern of actinotrichia changed from a planar layer at 9 dpf to a wavy layer (Fig. 4B). At 14 dpf, the late stage of the notochord bending, actinotrichia were multilayered and partially accumulated in bundles in the dorsal region (Fig. 4B).

Next, we used a photo-conversion system of actinotrichia to clarify whether they actually migrate to the dorsal region (Fig. 4C). In our previous study, we generated a Tg line that specifically expresses And1-KikGR in fins and established a system that changes the fluorescent color of actinotrichia by UV stimulation (41). Therefore, we next changed fluorescent colors of actinotrichia in various regions of the fins by photo-conversion and examined the distribution pattern of actinotrichia before and after the notochord bending. First, we performed photo-conversion at 6 dpf before the notochord bending at the base region of the fin on ventral side and changed fluorescence color of actinotrichia from green to red (Fig. 4D). At 12 dpf, the mid stage of the notochord bending, surprisingly, red fluorescent-labeled actinotrichia disappeared throughout the fins (Fig. 4D). Next, at 6 dpf, we changed fluorescent color of actinotrichia by photo-conversion on the midline of the fish body at the base region of the fin (Fig. 4D and SI Appendix, Fig. S7). As a result, their positions were shifted to dorsal side at 12 dpf (Fig. 4D and SI Appendix, Fig. S7). Next, we performed photo-conversion at the base region of the fin on the dorsal side and examined the distribution pattern of the actinotrichia labeled with red fluorescent (Fig. 4D and SI Appendix, Fig. S7). During the process of notochord bending, red-labeled actinotrichia were found to shift their position from fin base to more dorsal area (Fig. 4D and SI Appendix, Fig. S7). Furthermore, they also showed a change in alignment at 12 dpf, as if compressed in the anterior–posterior direction (Fig. 4D and SI Appendix, Fig. S7). These results indicate that actinotrichia dynamically change their original alignment pattern by moving dorsally in the process of notochord bending (Fig. 4E). In this photo-conversion system, it was difficult to sufficiently change the fluorescent color of actinotrichia in the fin tip region, so we could not examine the dynamics of actinotrichia in this region. We next decided to examine the dynamics of actinotrichia located in the tip region of the fins after the notochord bending by DAFFM labeling.

### Dynamics of actinotrichia migration towards distal fin tip during fin growth

**Fig. 5.**
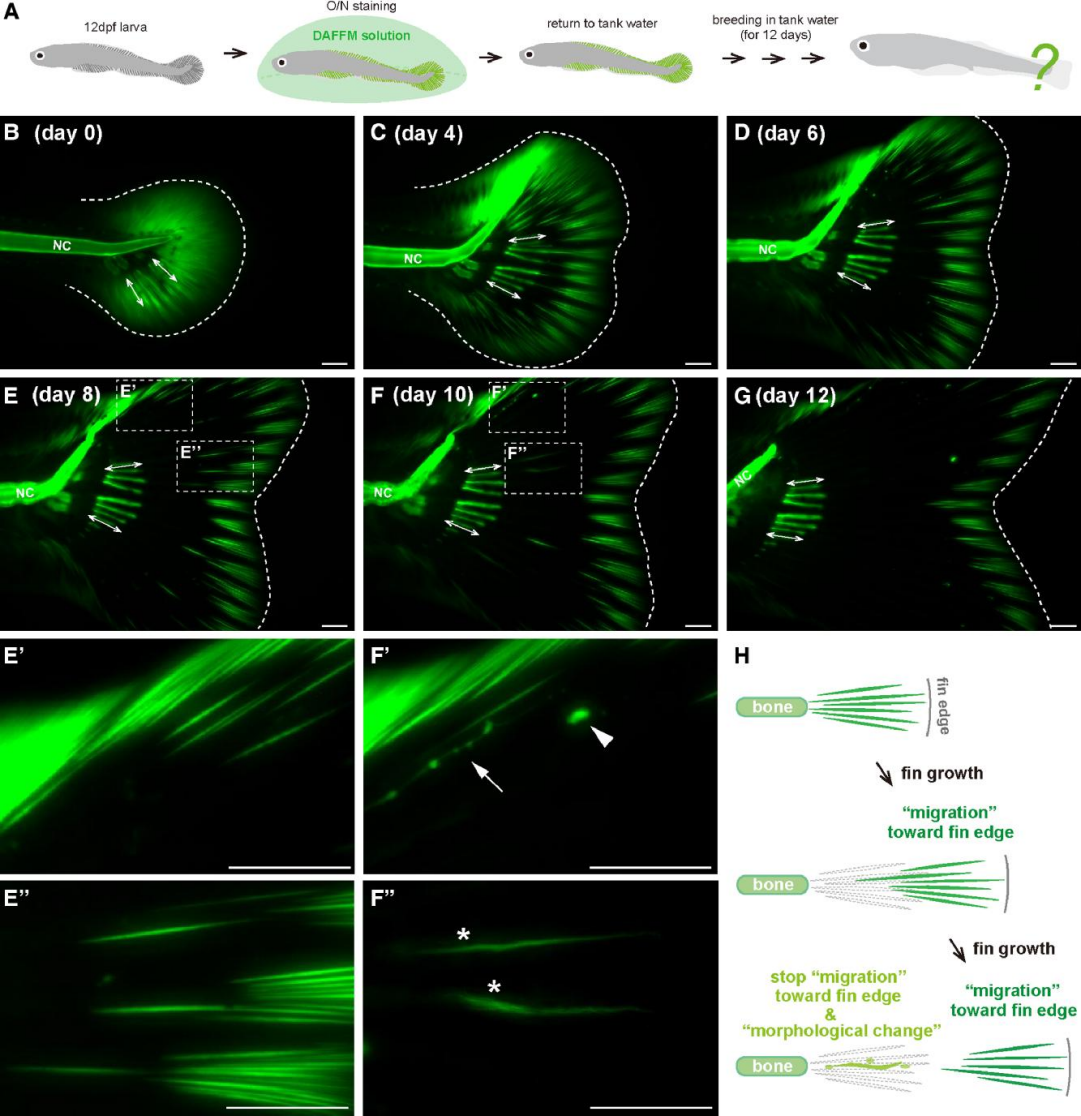
Dynamic movement of actinotrichia in the direction of distal fin tip during fin growth. (*A*) Workflow for the pulse-chase observation of actinotrichia by DAFFM staining after notochord bending. Living 12 dpf larvae were incubated overnight in DAFFM solution and then returned to tank water. After the staining, the distribution pattern of actinotrichia was observed over time during the 12 days of breeding. (*B* to *G*) The fluorescence images of the actinotrichia in the caudal fins at day 0 to day 12 after DAFFM staining. White dotted lines indicate outlines of the caudal fins in (*B*, *C*) and the tip of fins in (*D* to *G*). Double arrows indicate the fluorescent signals in fin rays formed at 12 dpf. NC, notochord. (*E’*, *E’’*) and (*F’*, *F’’*) enlarged views of the area in yellow dotted boxes in (*E*) and (*F*). Some actinotrichia exhibited different morphological changes, such as becoming small and oval (arrowhead in *F’*), thin and elongated (arrow in *F’*), or wavy in shape (asterisks in *F’’*). (*H*) Summary diagram of the actinotrichia migration towards distal fin tip during fin development. Scale bars, 100 μm.

As shown in Fig. 3, actinotrichia labeled by DAFFM at the early stage of fin formation were localized at the tips of the fins after the fins increased in size (Fig. 3BII). This suggests that actinotrichia move toward the distal tip of the fin during the fin growth. We next tested whether actinotrichia actually move in the direction of fin growth using a pulse-chase observation with DAFFM staining at a stage after the notochord bending (Fig. 5A). First, we treated 12 dpf larvae with DAFFM and labeled actinotrichia distributed throughout the fins, including the tips of the fins (Fig. 5B). The green fluorescence signals were also found in the fin rays at the early stage of bone formation, (Fig. 5B, double arrows). After the fins changed their shape to a fan shape, the distribution of DAFFM-labeled actinotrichia was found at the only tip region of the fins (Fig. 5C). In addition, we also found that the labeled actinotrichia continued to be distributed at the distal tip of the fins even while fins were actively elongating toward the distal direction (Fig. 5D–G). This fact suggests that once actinotrichia are formed, they do not stay stationary but instead continue to migrate towards the distal fin tip as the fins grow.

To investigate whether this novel dynamic of actinotrichia migration is conserved in the fin formation process in teleost fish other than zebrafish, we next examined actinotrichia dynamics during fin formation in medaka fish. On the day of hatching, medaka larvae were stained with DAFFM and returned to breeding water after staining (SI Appendix, Fig. S8). The results showed that actinotrichia in medaka's caudal fins shifted their distribution position toward the distal fin tip during fin growth (SI Appendix, Fig. S8). This result suggests that the dynamics of actinotrichia migration is a conserved morphogenetic event during fin formation in teleost fish.

In addition, we observed some newly positioned actinotrichia at distant areas from the base side of the actinotrichia bundle (Fig. 5E’ and E”). More interestingly, these actinotrichia shifted their arrangement toward the proximal side of the fins and significantly changed their morphology (Fig. 5F’ and F”). As shown in Fig. 3B III, there are various patterns of morphological changes in actinotrichia, and these actinotrichia that changed to irregular shapes completely disappeared from the fin tissues during the subsequent fin growth (Fig. 5G). We next attempted to perform time-lapse analysis using a confocal microscope to capture the morphological changes of actinotrichia in the fins of living fish. As a result, actinotrichia, which had a spear-like shape at the beginning of the imaging, dynamically changed its shape and gradually shifted the position in the proximal direction (Movie S3). These findings imply that actinotrichia constantly migrate in the direction of the distal fin tip during the fin growth (Fig. 5H) and also suggest that actinotrichia stop the migration at some point and are degraded by an unknown mechanism (Fig. 5H).

Furthermore, we asked whether the migration of actinotrichia to the distal fin tip is caused by expansion of the entire fin tissue. To investigate this possibility, we labeled mesenchymal cells around actinotrichia at the fin tip by photo-conversion and evaluated their migration patterns (SI Appendix, Supplementary Material text S3 and Fig. S9). As a result, we found that mesenchymal cells exhibited different migration patterns depending on the fin areas (details are shown in SI Appendix, Supplementary Material text S3 and Fig. S9). These results indicate that the “actinotrichia migration” toward the distal tip of fins is not simply caused by the expansion and growth of the entire distal fin tissues.

### Growth dynamics of actinotrichia revealed by pulse-chase observation using two different probes

A previous study reported that actinotrichia increase in size during fin growth (50). Furthermore, as shown in Fig. 5, some actinotrichia presumably undergo degradation as they move toward the distal fin tip. This process may reduce the density of the actinotrichia bundle at the fin tip, but it should maintain a constant density throughout fin growth. To investigate how actinotrichia grow at the fin tip, we performed pulse-chase experiments using two types of fluorescent probes, DAFFM and DAR4M, at different time points. As shown in Fig. 2, the fluorescence of actinotrichia stained with DAFFM did not fade after 40 days of fish breeding, allowing us to examine the growth pattern of actinotrichia by staining them with DAR4M after a long period of fish breeding.

**Fig. 6.**
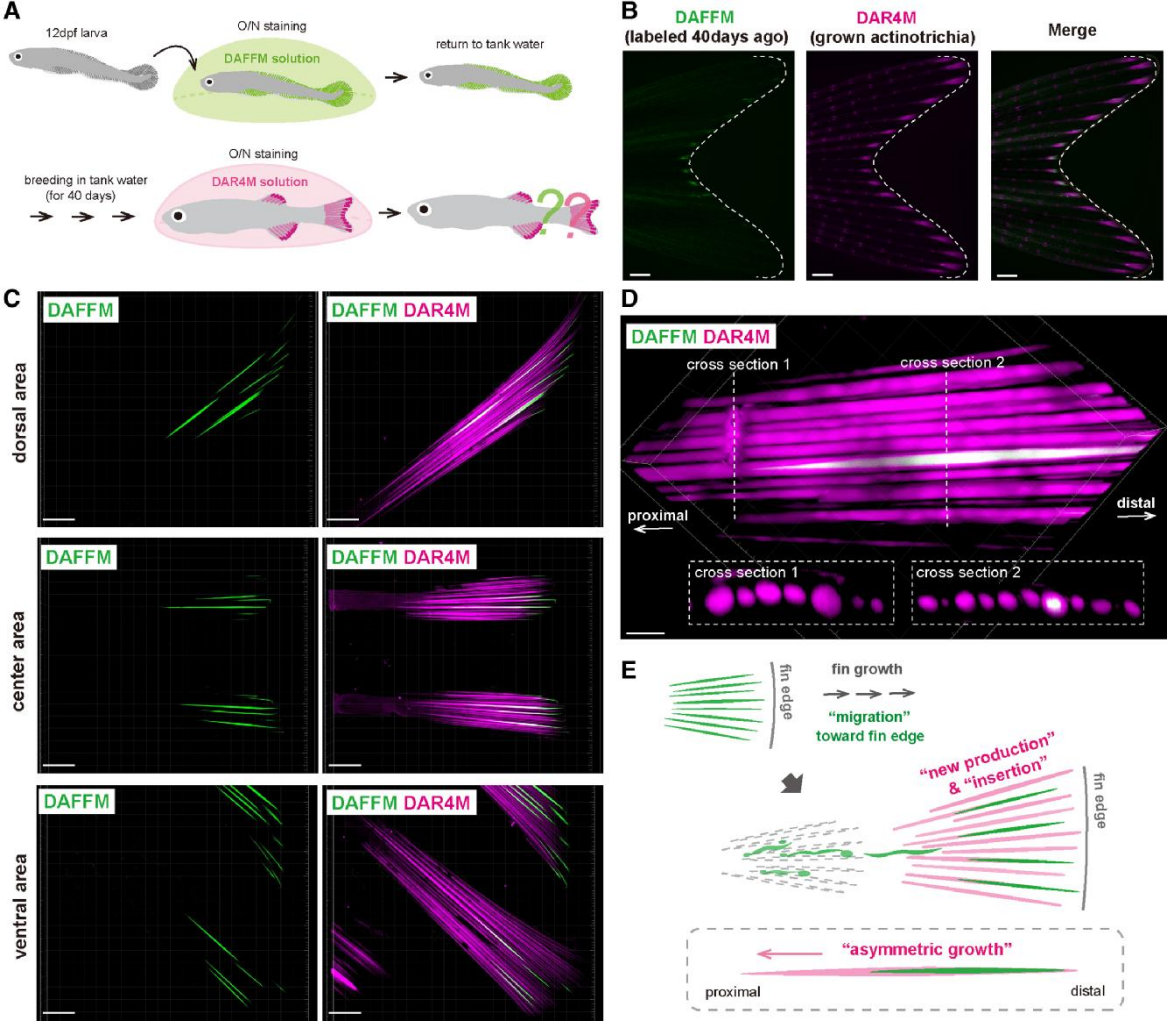
Growth dynamics of actinotrichia bundle. (*A*) Procedure for the pulse-chase observation of actinotrichia using DAFFM and DAR4M. (*B*) Fluorescent images of the caudal fin stained with DAF and DAR at different stage. White dotted lines indicate the distal margin of the caudal fin. (*C*) Confocal images of the actinotrichia bundles around the dorsal, center, and ventral areas at the distal fin tip. (*D*) A higher magnification confocal image of the actinotrichia bundle around ventral area at the distal fin tip. Cross-sectional views at white dotted lines 1 and 2 are shown on lower panels. (*E*) Summary diagram of the growth dynamics of actinotrichia bundle during fin growth. Scale bars, 400 μm (*B*), 50 μm (*C*), and 20 μm (*D*).

We first stained 12 dpf larvae at the notochord bending stage with DAFFM and then returned them to tank water for 40 days of breeding. After 40 days, we stained the actinotrichia with DAR4M (Fig. 6A). During this period, the caudal fin changed to an M-shape with dorsal and ventral projections due to the active growth of these regions (Fig. 6B). We examined the growth patterns of actinotrichia at the tips of these M-shaped fins for each of the three regions: dorsal, central, and ventral. In the dorsal region of the fin tip, we observed short DAFFM-labeled actinotrichia (<100 μm), which were labeled 40 days ago, within the long DAR4M-labeled actinotrichia (Fig. 6C and Movie S4). Interestingly, the DAFFM fluorescence was located on the distal side of each actinotrichia. This indicates that the actinotrichia grow anisotropically by mainly extending toward the proximal side, rather than isotropically in both directions, while moving toward the distal fin tip. Notably, the old DAFFM-labeled actinotrichia were sparsely distributed in the actinotrichia bundles, and the new DAR4M-labeled actinotrichia filled the gaps between them (Fig. 6C and Movie S4). We also observed DAFFM fluorescence on the distal side of each actinotrichia in the central region of the fins (Fig. 6C). However, the length difference between DAFFM- and DAR4M-labeled actinotrichia was smaller than that in the dorsal region (Fig. 6C). We also observed anisotropic growth and new actinotrichia insertion in the ventral region, similar to the dorsal region (Fig. 6C). Additionally, in the magnified cross-sectional image, we detected no DAFFM fluorescence on the proximal side of the actinotrichia, neither on the surface nor inside, but only DAR4M fluorescence (Fig. 6D). On the distal side, however, we found DAFFM fluorescence within the DAR4M fluorescent region in the cross-sectional image (Fig. 6D). This result indicates that actinotrichia move toward the distal fin tip, thicken by adding new collagen to their surface, and further extend in the proximal direction.

### Dynamics of actinotrichia degradation induced by osteoclasts

As shown in Fig. 3B III and Fig. 5F, some actinotrichia changed into various irregular shapes during fin growth. Furthermore, these actinotrichia disappeared completely within a few days (Fig. 5F and G). During this characteristic morphological change of actinotrichia, we observed that the actinotrichia seemed to be “dissolved”. Previous studies have shown that osteoclasts are the cells that dissolve large ECM structures in vertebrates (51–54). Osteoclasts are known to adhere to bone matrix using podosomes, which are characteristic structures of the actin cytoskeleton, and play a role in degrading and digesting bone matrix including collagen (55–57). Therefore, we hypothesized that osteoclasts actively degrade actinotrichia during fin growth.

**Fig. 7.**
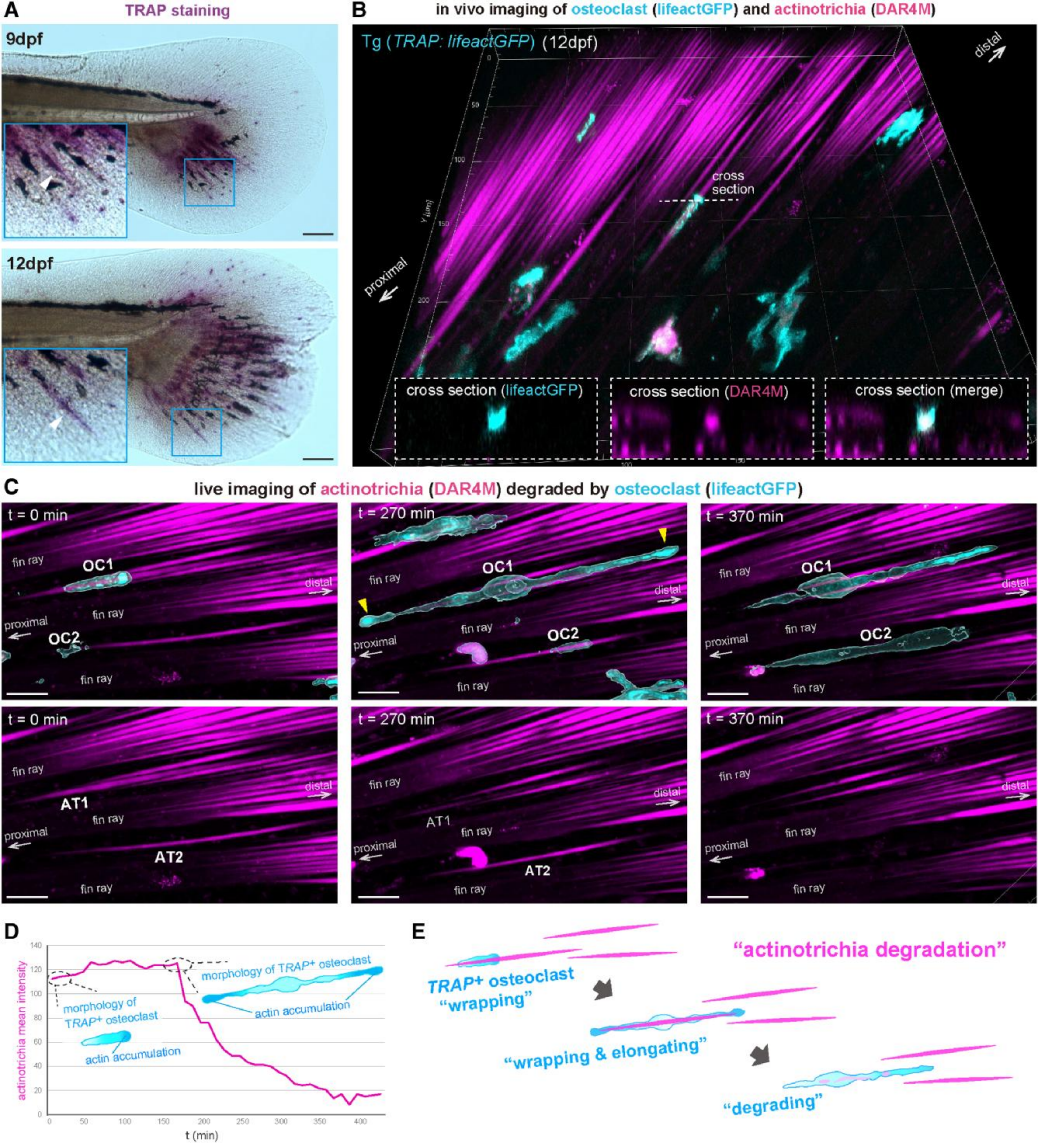
Degradation dynamics of actinotrichia induced by the physical interaction with osteoclasts. (*A*) Bright field images of the larval caudal fins after TRAP staining. TRAP activities of osteoclasts are indicated by purple coloration. Specific coloration was observed in regions with size and shape comparable to actinotrichia (white arrowhead in blue box). (*B*) In vivo fluorescent imaging of *TRAP*-expressing osteoclasts (cyan) and actinotrichia (magenta). Actinotrichia in Tg (*TRAP*:*lifeactGFP*) larval caudal fins were labeled with DAR4M. Cross-sectional views at white dotted line are shown on lower panels. (*C*) In vivo live imaging of the interaction between osteoclasts and actinotrichia. 3D confocal images of osteoclasts (cyan) and actinotrichia (magenta) at each time point (*t* = 0, 270 and 370 min) are shown. A single osteoclast (OC1, OC2) interacted with a single actinotrichia (AT1, AT2) (*t* = 0 min) and gradually elongated along actinotrichia as accumulating actin (yellow arrowheads) on both their proximal and distal sides (*t* = 270 min). AT1 started to be degraded during the 270 min after the start of time-lapse imaging and AT2 was degraded during the following 100 min. (*D*) Plot profile of the fluorescent intensity at AT1 shown in (C). The fluorescent level began to decrease around the time point (*t* = 150 min) when the osteoclast fully extended in length. (*E*) Schematic illustration of the actinotrichia degradation by *TRAP*-expressing osteoclast during fin development. Scale bars, 100 μm (*A*) and 20 μm (*C*).

First, we performed tartrate-resistant acid phosphatase (TRAP) staining, which allows for a simple examination of osteoclast activity (Fig. 7A). As a result, we confirmed strong staining in the ventral region of the fins at 9 dpf, the early stage of notochord bending (Fig. 7A). Interestingly, we found the staining pattern very similar to actinotrichia shape (Fig. 7A, blue box). In addition, we also detected this characteristic staining pattern at 12 dpf (Fig. 7A, blue box). These results suggest that osteoclasts with TRAP activities are involved in the degradation of actinotrichia in fins.

Therefore, we next-generated transgenic zebrafish expressing *lifeact-gfp* under the *TRAP* promoter to visualize the actin cytoskeleton of osteoclasts and investigated the dynamics of the interaction between osteoclasts and actinotrichia. In this analysis, we stained the Tg fish using DAR4M to label actinotrichia with red fluorescence. As a result, we observed *TRAP*-expressing osteoclasts elongating along the actinotrichia axis (Fig. 7B and Movie S5). In addition, cross-sectional images showed that osteoclasts physically interacted with actinotrichia by extending actin-rich domains that enveloped them (Fig. 7B, cross-section).

We hypothesized that osteoclasts with this characteristic morphology had the ability to degrade actinotrichia. In vivo live imaging of the physical interaction between osteoclasts and actinotrichia revealed that osteoclasts actually degraded actinotrichia (Fig. 7C, SI Appendix, Figs. S10–S13 and Movies S6–S10). When osteoclasts initially attached to actinotrichia located in the interray space, they were compact in size (Fig. 7C, *t* = 0 min). However, as they wrapped actinotrichia, they started to elongate along the long axis of the actinotrichia. In this process, osteoclasts formed actin-rich structures at their proximal and distal ends (Fig. 7C, *t* = 270 min yellow arrowheads). After these dynamic morphological changes of the osteoclasts, the structures of actinotrichia quickly disappeared (Fig. 7C, *t* = 370 min). In addition, we found that the osteoclasts, which display distinctive dynamic morphological changes, cause the degradation of actinotrichia not only in the interray but also in other areas of the caudal fin (SI Appendix, Figs. S10–S13 and Movies S7–S10). These results indicate that actinotrichia indeed undergo degradation during fin growth and disappear from the fin tissue with dynamic changes in their shapes. Furthermore, it appears that *TRAP*-expressing osteoclasts play a central role in this degradation process.

## Discussion

We found that DAFFM/DAR4M, previously known as detection probes for nitric oxide (NO) (48, 49, 58), can fluorescently label zebrafish actinotrichia with a simple method. These probes are extremely useful for the pulse-chase observation during fin growth because of their low toxicity and nondecreasing fluorescence intensity. Furthermore, since DAFFM and DAR4M have different fluorescence wavelengths (48, 49), it is possible to distinguish and track-specific actinotrichia. Using this approach, we found that actinotrichia exhibit novel and multiple unexpected dynamics during fin growth. A particularly interesting dynamics is the actinotrichia migration along the ventral–dorsal and proximal–distal axis of the fins. This fact sheds new light on the understanding of the mechanism of fin formation, since actinotrichia had previously been perceived as “immobile structures”. The insertion of newly produced actinotrichia at the growing ends of fins and the site-specific degradation of actinotrichia by osteoclasts are also important novel findings. We predict that our method will become the standard for actinotrichia studies in the future. Although DAFFM/DAR4M are extremely useful for analysis of actinotrichia dynamics in live fish, they label all actinotrichia within a specific time frame. Therefore, it is difficult to reveal the dynamics of individual actinotrichia in a specific region using these probes. For more detailed analysis of the dynamics of individual actinotrichia, it is necessary to combine other labeling approaches including photo-conversion experiments.

DAFFM and DAR4M stain actinotrichia very clearly in zebrafish fins, but the mechanism behind this staining is currently unknown. The two molecules have been widely used as fluorescent probes for the detection of intracellular NO in various types of cells (59). Previous studies reported that DAFFM can stain bones and cartilaginous tissues in zebrafish, and also suggested that NO is involved in the fluorescent staining of these tissues in zebrafish with DAFFM (42–44). However, our results in the current study showed that NO do not play a role in the fluorescent staining of actinotrichia (SI Appendix, Supplementary Material text S2 and Fig. S2). Recently, Nasuno and colleagues reported that DAFFM emits fluorescence upon binding to aldehyde residues, independent of NO (60), and this effect may explain the fluorescent labeling of actinotrichia. Collagen fibrils form large fibrous structures by self-assembly of three chains of collagen molecules (2, 4, 61), and each collagen fibril is cross-linked by covalent bonds between collagen molecules (2, 62). The chemical reaction for the formation of cross-linking is catalyzed by LOX (lysyl oxidase), and an aldehyde is formed as a temporary intermediate of the cross-linking (63–65). Since actinotrichia is a structure of collagen fibrils composed of a large number of collagen molecules, it is not surprising that it contains a large number of aldehydes. If DAFFM binds to the intermediate structure of collagen cross-linking, it could be used for fluorescent labeling of collagen fibers other than actinotrichia. In fact, we confirmed that tissues rich in collagen fibers, such as the notochords and bones, are also fluorescently labeled by DAFFM (as shown in Figs. 1B and 5B–G). If other derivatives of DAFFM can be screened to find more sensitive reagents, they could be used as fluorescent staining reagents for finer collagen fibers.

The structure of an adult body appears unchanged and stable on a macroscopic scale. However, on a cellular microscopic scale, the shape is maintained through constant scrap building. Therefore, the shape can be explained as a state of equilibrium created by the balance of multiple dynamic elementary processes. This is also the case with the actinotrichia in fins. Actinotrichia are always aligned in the same density at the fin tip and maintain radial orientation, with bundle formation corresponding to the branching of fin rays. Considering that the distal tip region of fins continues to grow, it is essential that actinotrichia continue to change on a microscopic scale to maintain this state. In the current study, we clarified the various novel dynamics of actinotrichia during fin growth. Namely, “migration toward the distal fin tip to maintain positioning at the fin tip region”, “insertion to maintain uniform density”, “elongation to the proximal side”, and “selective degradation which contributes to the formation of bundling pattern”. Further investigation into the details of how and which cells achieve these elementary processes will advance our understanding of fin formation. Although actinotrichia are unique collagen structures containing ECM factors of unknown function, Actinodin family proteins, their main components, type I and type II collagen fibers, are highly conserved among vertebrate animals. The structural integrity and morphology of animal organs are intrinsically linked to the proper organization of these collagen fibers. Thus, findings on fin formation are likely to contribute to organogenesis in vertebrates that do not have fins.

## Materials and methods

Zebrafish were maintained under the standard laboratory conditions and treated as previously described (66). AB strains were used as wild type zebrafish. The lines were used in this study: *Tg* (*col2a1a: H2B-mRFP*), *Tg* (*and1 1.4k: And1^480bp^-KikGR*) (41), *Tg* (*5xand1*(*MC*): *KikGR*), *Tg* (*5×and1*(*MC*): *lifeact-mCherry*) (41), *Tg* (*osx: lifeact-mCherry*) (67), *Tg* (*3×osx: lifeact-YFP*) (26), *Tg* (*TRAP: lifeact-GFP*). These Tg lines were generated by injecting the tol2 plasmid with Tol2 transposase (68). Japanese medaka (Oryzias latipes) were bred and maintained under the conditions with 14 and 10 h of light (8:30 AM–10:30 PM) and dark cycles at 25°C. They were fed commercial powder food 2 times a day. All zebrafish and medaka experiments were approved by the animal care and use at Osaka University.

A complete description of Materials and methods is available in the SI Appendix, Materials and Methods.

## Supplementary Material

pgae266_Supplementary_Data

## Data Availability

All data are included in the manuscript and Supplementary Material.
