# Supplementary material for: Dynamics of actinotrichia, fibrous collagen structures in zebrafish fin tissues, unveiled by novel fluorescent probes
Source: PNAS Nexus. 2024 Jul 5;3(7):pgae266. doi: 10.1093/pnasnexus/pgae266 (PMC11409509; doi:10.1093/pnasnexus/pgae266)
Supplement: pgae266_Supplementary_Data [file pgae266_supplementary_data.zip › PNASNEXUS-PNASNEXUS-2024-00053-TR-s01.pdf]

PNAS nexus

Supporting Information for
Dynamics of actinotrichia, fibrous collagen structures in zebrafish fin tissues, unveiled by novel fluorescent probes

Junpei Kuroda[1]*, Hiromu Hino[1] and Shigeru Kondo[1].
[1] Graduate School of Frontier Bioscience, Osaka University, Suita, Japan.

* Corresponding author: Junpei Kuroda.
Email: jkuroda@fbs.osaka-u.ac.jp

This PDF file includes:

Supporting text S1 to S3
Figures S1 to S13
Legends for Movies S1 to S10
SI Materials and Methods
SI Appendix References

Other supporting materials for this manuscript include the following:

Movies S1 to S10

Supporting text S1.

In previous studies, DAFFM solution was adjusted to a concentration of 5 μM and used for staining (1, 2), so we evaluated the actinotrichia fluorescence in larval fins using this method. Living juvenile fish at 5 days post fertilization (dpf) were incubated in DAFFM solution adjusted with breeding water and treated at room temperature for 1 hr, 2 hr, 4 hr, 6 hr, O/N (12 hr). The fluorescence intensity of actinotrichia was highest when the fish were treated with O/N (12 hr) (*SI Appendix*, Fig. S1). Next, we tested whether DAR4M can fluorescently stain actinotrichia. Living 5 dpf larvae were incubated in DAR4M solution adjusted to a concentration of 5 μM and stained for various times at room temperature. Weak red fluorescence was observed in the notochord, while almost no fluorescence was observed in the actinotrichia (data not shown). Furthermore, when staining was performed at an increased concentration of 10 μM, the fluorescence intensity of the notochord increased, but almost no fluorescence of actinotrichia was observed even at this concentration (data not shown). Next, young fish at the stage of fin ray formation (3 weeks age) were incubated in DAR4M solutions at concentrations of 5 or 10 μM, and the fluorescence intensity of actinotrichia was examined. As a result, actinotrichia emitted the strongest fluorescence under the conditions of the incubation in 10μM solution O/N (12 hr) (*SI Appendix*, Fig. S3).

Supporting text S2.

DAFFM is widely used as a detection probe for nitric oxide (NO). Additionally, we have previously reported that in zebrafish fins, mesenchyme cells develop long pseudopodia and physically contact with the surface of actinotrichia. Therefore, it was hypothesized that the fluorescent emission of actinotrichia by DAFFM treatment might be caused by NO released by mesenchymal cells. To investigate this hypothesis, we isolated actinotrichia from fin tissues and stained them with DAFFM in a state of loss of influence from surrounding cells. Remarkably, the isolated actinotrichia were distinctly labeled with DAFFM *in vitro* (*SI Appendix*, Fig. S2). Next, to further validate the involvement of NO, actinotrichia were stained with DAFFM under conditions of inhibition of the nitric oxide synthase (NOS) activity, and under conditions of NO removal. As a result, the isolated actinotrichia were distinctly labeled with fluorescence under these conditions. Furthermore, we found that the fluorescence intensity of actinotrichia labeled with DAFFM under conditions of NOS inhibition or NO removal was unchanged from that under conditions without these drug treatments (*SI Appendix*, Fig. S2). These results indicate that NO does not play a role in the fluorescent labeling of actinotrichia by DAFFM.

Supporting text S3.

There are two possible mechanisms by which the labeled actinotrichia remain constantly distributed in the distal tip of the fins during fin growth. The first possibility is that the tissue at the tips of the fins is simultaneously expanding and growing in clusters. The second possibility is that actinotrichia are constantly moving in the direction of fin growth. In the former case, actinotrichia and the surrounding cells should always show a similar movement pattern and shift their arrangement in the growth direction as the fins grow. In the latter case, on the other hand, the migration speed or directionality of actinotrichia and cells should not coincide, and a more active shift of actinotrichia placement in the fin distal direction should be observed. Previous studies have shown that the actinotrichia at the tips of the fins are surrounded by elongated pseudopodia of mesenchymal cells (3, 4). We then fluorescently labeled actinotrichia and mesenchymal cells in the distal tip of the fins and compared their dynamics during the fin growth to elucidate the manner of "actinotrichia migration". We treated F0 fish of Tg (*and1(MC) pro: KikGR*), which express KikGR in a mosaic specifically in the mesenchymal cells of the fins, with DAFFM for the actinotrichia labeling (*SI Appendix*, Fig. S9). We labeled mesenchymal cells with red fluorescence by photo-conversion in three regions (tip, middle, and base at the actinotrichia bundle), and examined changes in their distribution during fin growth together with actinotrichia labeled with

green fluorescence (*SI Appendix*, Fig. S9). Interestingly, we found that mesenchymal cells exhibited different dynamics in each region. Firstly, mesenchymal cells located in the tip region of the actinotrichia shifted their arrangement in the distal tip direction while elongating along the actinotrichia during the 6-day growth (*SI Appendix*, Fig. S9). Secondly, the mesenchymal cells located near the center of the actinotrichia bundle, while elongating along the actinotrichia, split into two cells during the fin growth, and one was positioned at the base of the actinotrichia bundle (*SI Appendix*, Fig. S9). And thirdly, mesenchymal cells located in the base region of the actinotrichia continued to maintain their original position while the actinotrichia shifted their arrangement toward the distal tip region (*SI Appendix*, Fig. S9).

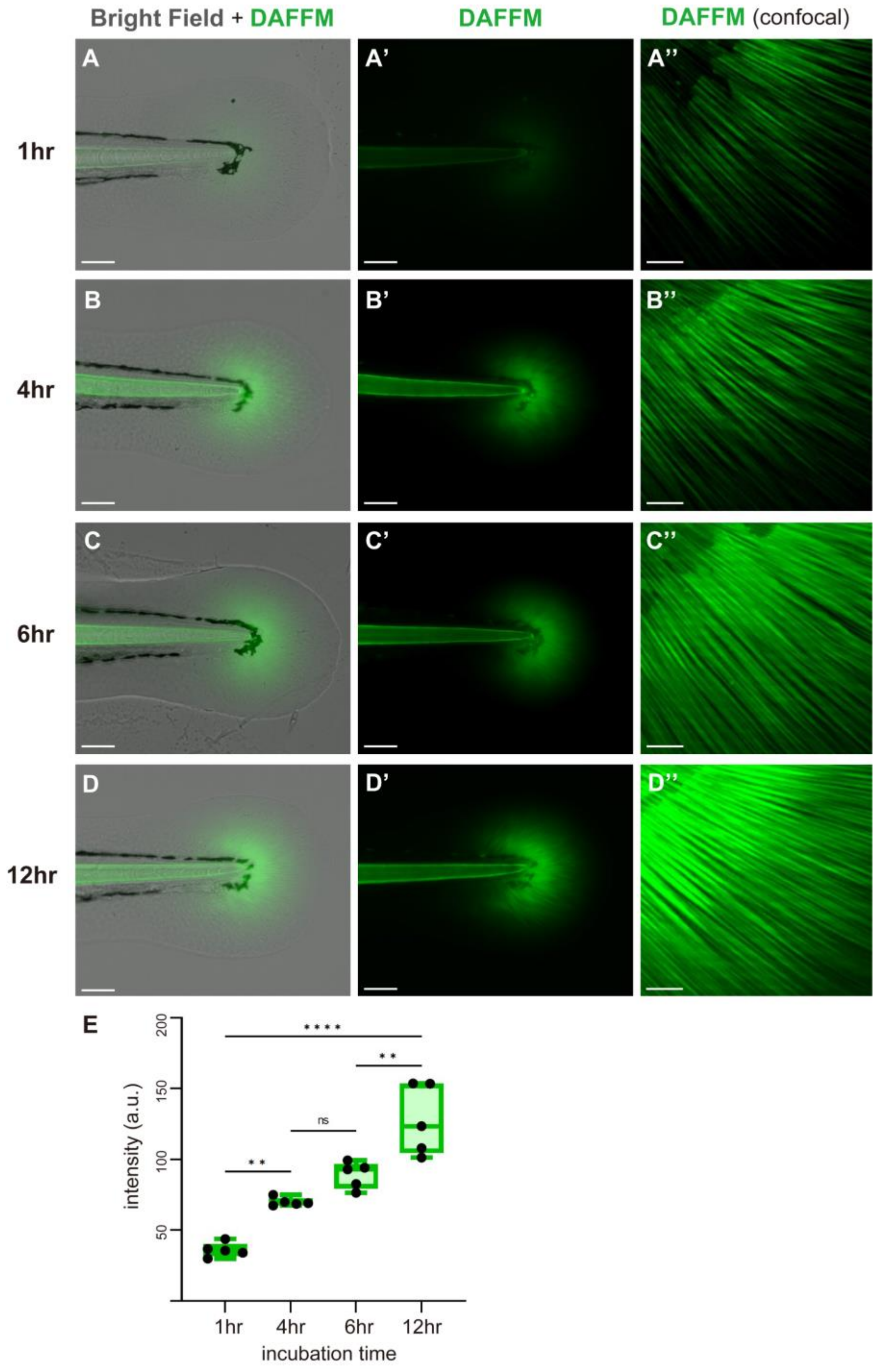

Fig. S1. Fluorescence intensity of actinotrichia after different treatment durations with DAFFM**.** Living larval zebrafish at 5dpf were incubated in the DAFFM 5μM solution at room temperature. Fluorescence images of the entire caudal fins were taken after DAFFM staining for 1 hr (*A*-*A''*), 4 hr (*B*-*B''*), 6 hr (*C*-*C''*) and 12 hr (*D*-*D''*). (*A''*-*D''*) Actinotrichia around the ventral center region of each caudal fins were observed by a confocal microscopy. (*E*) The fluorescence intensity of actinotrichia was measured at each staining time. Scale bars, 100 μm (*A*, *A'*-*D*, *D'*) and 20 μm (*A''*-*D''*).

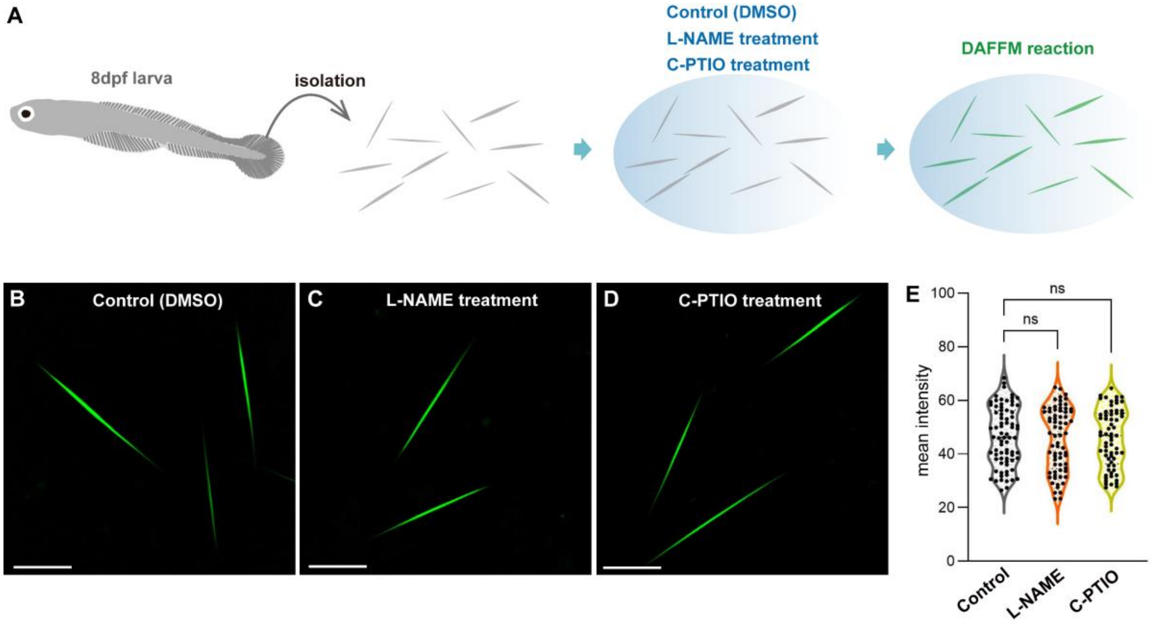

Fig. S2. Fluorescence visualization of actinotrichia by DAFFM not dependent on nitric oxide (NO). (*A*) Workflow for DAFFM staining of the isolated actinotrichia under the drug treatment conditions. After isolation from larval fins, actinotrichia were treated with three different conditions: control (DMSO), L-NAME (a NOS inhibitor) and C-PTIO (a NO remover), respectively. Under each condition, the actinotrichia were incubated with DAFFM solutions containing the corresponding drug. (*B-D*) Fluorescence images of the isolated actinotrichia visualized with DAFFM under control (DMSO), L-NAME and C-PTIO treatment conditions, respectively. (*E*) Fluorescence intensity of the actinotrichia visualized with DAFFM under three treatment conditions. There is no significant difference in fluorescence intensity under each condition, suggesting that NO is not involved in the fluorescence emission by DAFFM in actinotrichia. Scale bars, 50 μm.

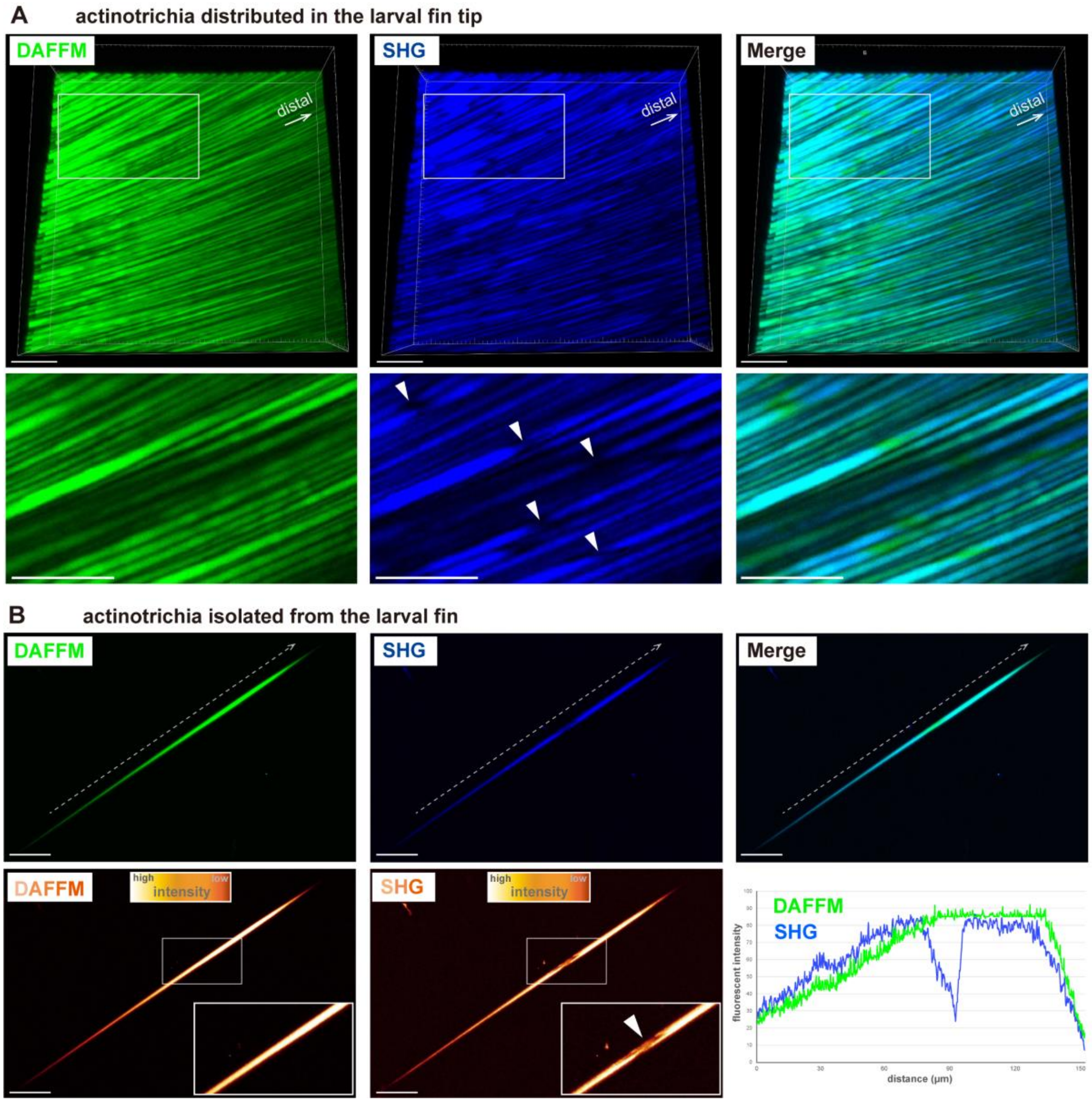

Fig. S3. Comparison of DAFFM and SHG fluorescence. Living larval zebrafish at 8dpf were incubated overnight in the DAFFM 5μM solution at room temperature. (*A*) Fluorescence images of the actinotrichia at the tip of the caudal fin observed with a two-photon microscope. The magnified images of single sections within the white box areas of each fluorescence image are shown in the lower panels, respectively. DAFFM clearly and uniformly visualized the shapes of actinotrichia, while SHG fluorescent signals was ununiform and partially undetectable in actinotrichia (indicated by white arrowheads). (*B*) Fluorescence images of the actinotrichia isolated from the larval fins after DAFFM staining. DAFFM fluorescence was observed throughout the actinotrichia, and its intensity was stronger in the thicker regions of actinotrichia. On the other hand, SHG fluorescence was partially indistinct in the actinotrichia (indicated by white arrowhead). Plot profiles of the fluorescent intensity (DAFFM and SHG) at the isolated actinotrichia are shown. Scale bars, 20 μm.

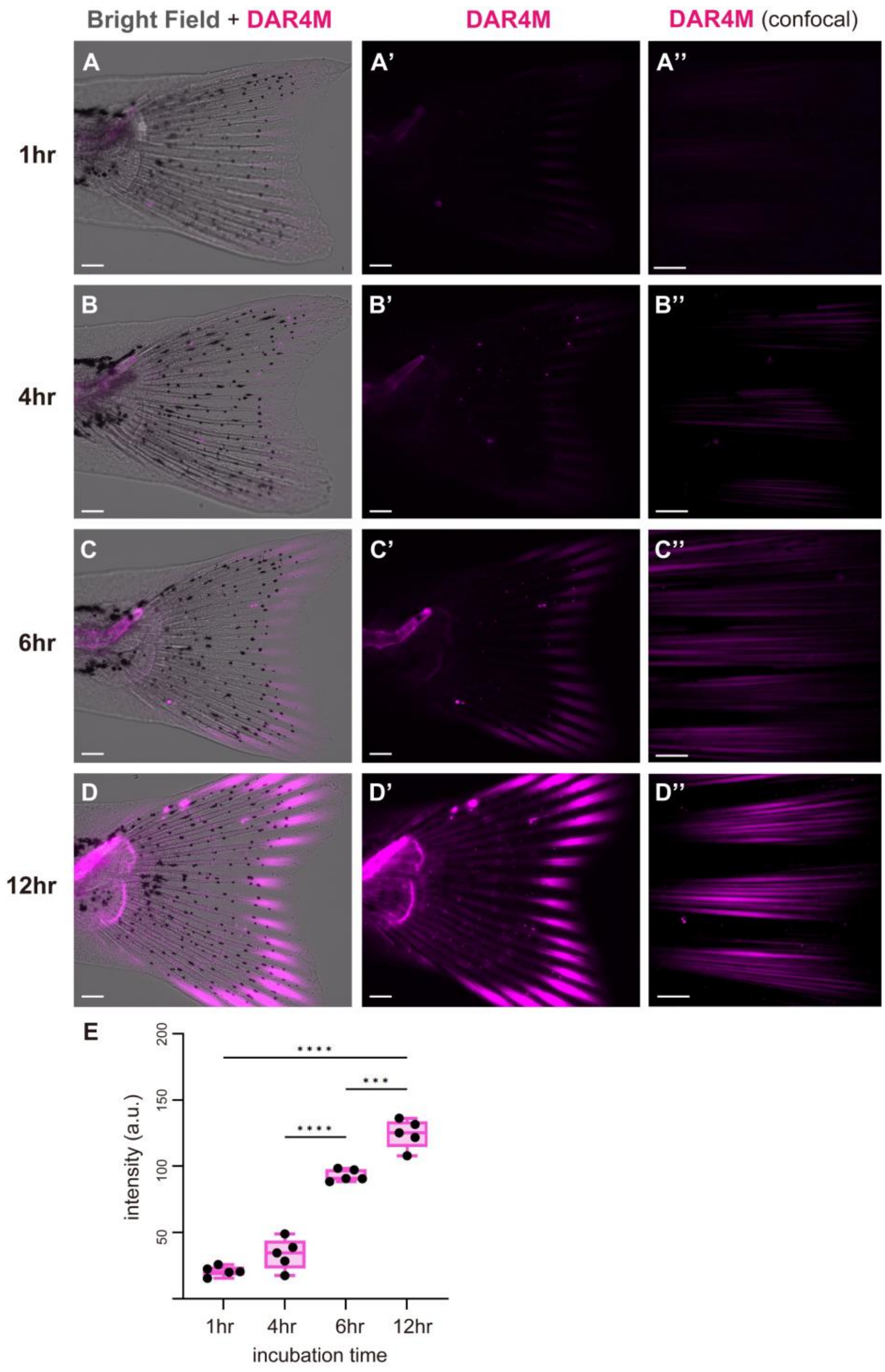

Fig. S4. Fluorescence intensity of actinotrichia after different treatment durations with DAR4M**.** Living larval zebrafish at 21dpf were incubated in the DAR4M 10μM solution at room temperature. Fluorescence images of the entire caudal fins were taken after DAR4M staining for 1 hr (*A-A''*), 4 hr (*B-B''*), 6 hr (*C-C''*) and 12 hr (*D-D''*). Actinotrichia located at distal tip in each caudal fins were observed by a confocal microscopy (*A''-D''*). The fluorescence intensity of actinotrichia was measured at each staining time (*E*). Scale bars, 100 μm (*A*, *A'-D*, *D'*) and 20 μm (*A''-D''*).

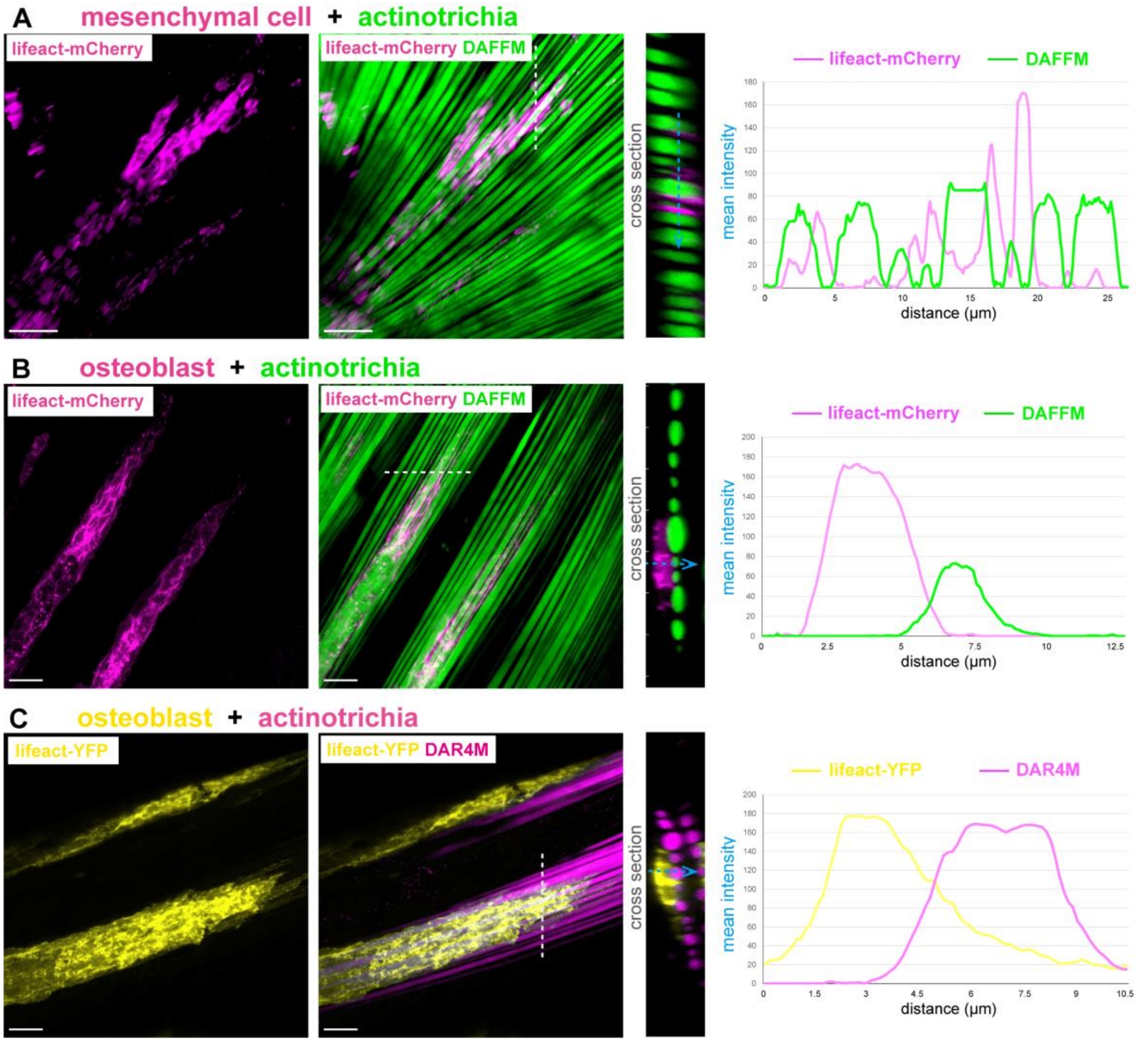

Fig. S5. Fluorescence imaging of actinotrichia visualized by DAFFM/DAR4M, combined with fluorescence of cell types distributed in the fins. (*A*) Simultaneous fluorescence observation of mesenchymal cells expressing lifeact-mCherry in F0 larval fish and actinotrichia labeled with DAFFM. (*B*) Simultaneous fluorescence observation of osteoblasts expressing lifeact-mCherry and actinotrichia labeled with DAFFM at tip of fin bones. (*C*) Simultaneous fluorescence observation of osteoblasts expressing lifeact-YFP and actinotrichia labeled with DAR4M at tip of fin bones. Each cross-sectional view at the white dotted lines in (*A*-*C*) is shown. The results of the intensity plot profile (shown in right panels) at the blue dotted line in each cross-sectional image show clear separation of the fluorescence peaks of the actinotrichia labeled with DAFFM/DAR4M and the cells expressing lifeact-mCherry/lifeact-YFP. Scale bars, 20 µm.

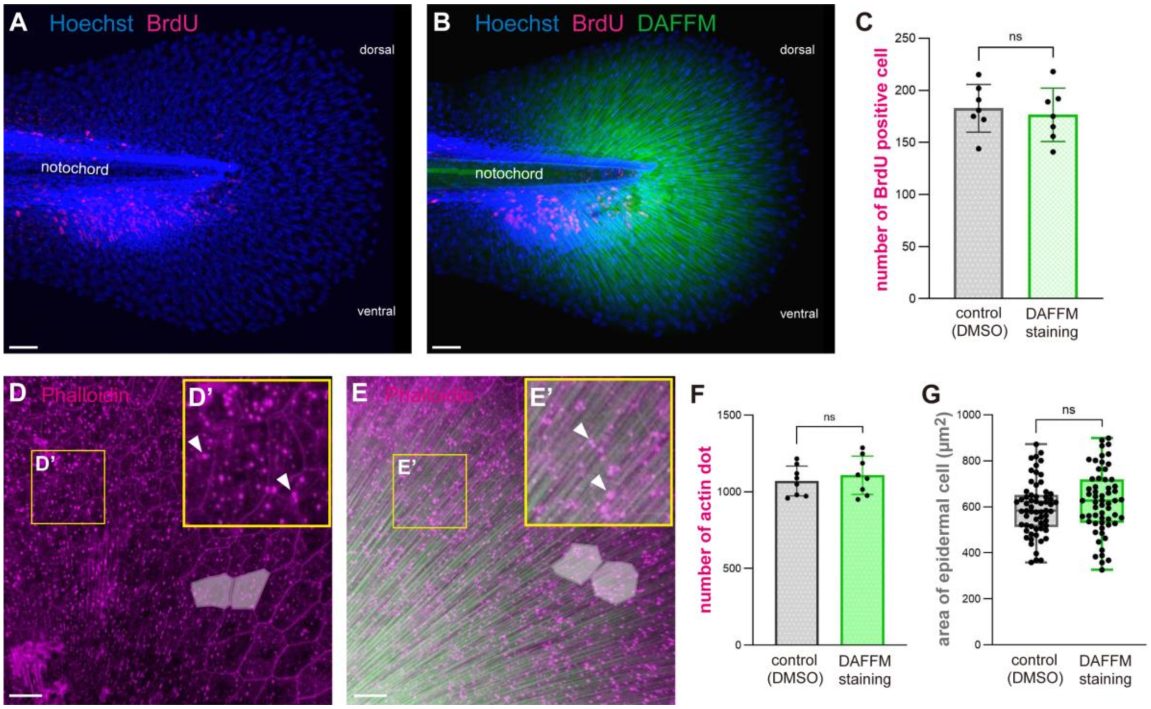

Fig. S6. No negative effects against cell activities with DAFFM treatment for living fish. (*A* and *B*) Confocal images of caudal fins in larvae at 6dpf. Larvae were incubated with DMSO (control, *A*) or DAFFM solution (*B*). All cell nuclei were stained with Hoechst (blue). Cell nuclei incorporating BrdU were stained with anti-BrdU antibodies (magenta). Actinotrichia were visualized with DAFFM (green). In both control and DAFFM-treated fins, BrdU positive cells were dense in the ventral region of the notochord. (*C*) The number of BrdU positive cells was counted under each condition. No significant difference was observed in the number of BrdU positive cells across the conditions. (*D* and *E*) Confocal images of caudal fins at dorsal area in larvae at 6dpf. Both DMSO (control, *D*) and DAFFM-treated fins (*E*) were stained with Phalloidin (magenta). Actinotrichia were visualized with DAFFM (green). In both conditions, numerous dot-shaped accumulations of actin (as reported in a previous study (5);white arrowheads in *D'* and *E*') were observed. (*F*) The number of actin dots was counted under each condition. There was no significant difference in the number of actin dots across the conditions. (*G*) Area of epidermal cells (as shown in white shading in *D* and *E*) was calculated under each condition. There was no significant difference in the area of epidermal cells across the conditions. Scale bars, 50 μm (*A* and *B*) and 20 μm (*D* and *E*).

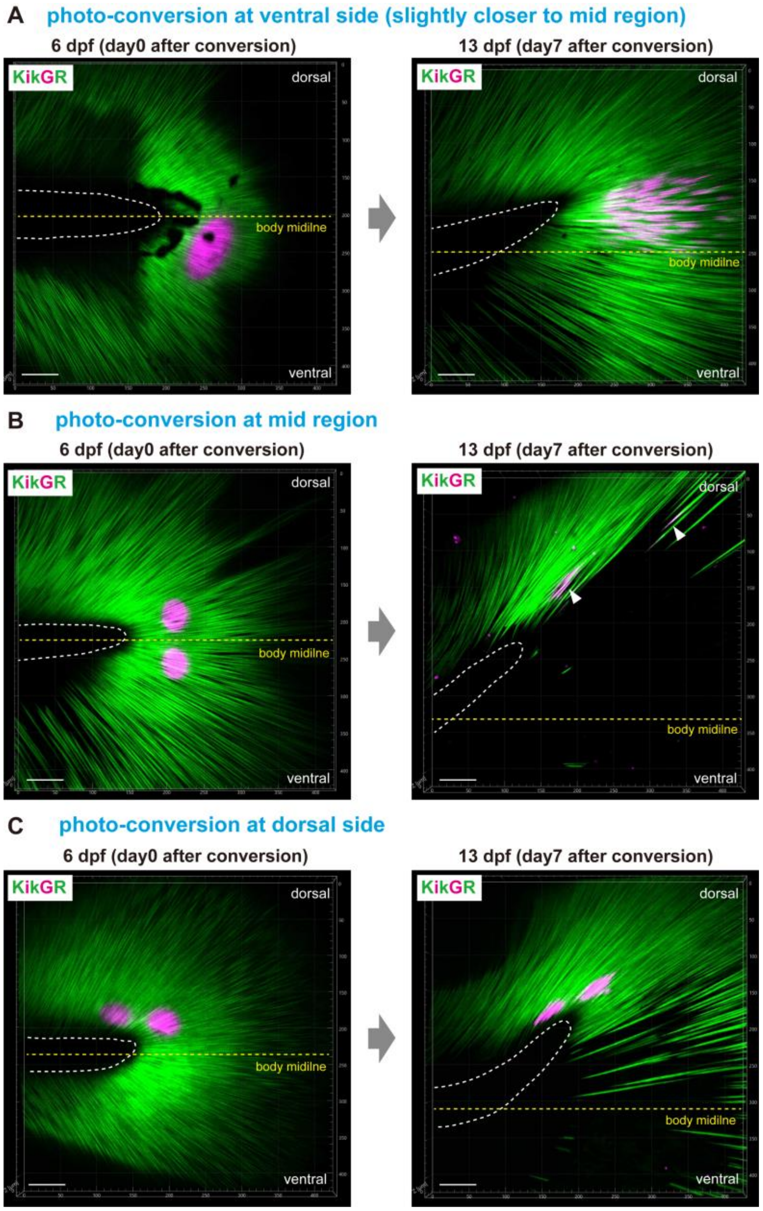

Fig. S7. Actinotrichia dynamic movement during notochord bending. Fluorescent colors of the actinotrichia in 6dpf larval fins visualized with And1-KikGR tagging were changed by UV stimulation at various fin regions, and the distribution patterns of the labeled actinotrichia were examined during notochord bending. (*A*) The fluorescent colors of actinotrichia were changed by the photo-conversion at ventral side slightly closer to mid region. On day 7 after photo-conversion, the positions of the labeled actinotrichia shifted to the dorsal side, and some of them also shifted to the distal side. (*B*) The fluorescent colors of actinotrichia were changed by the photo-conversion at two regions of the dorsal and ventral side near the body midline. On day 7 after photo-conversion, the positions of the labeled actinotrichia shifted to the dorsal side, and they were divided into two areas: the proximal area and the distal area (indicated by white arrowheads). (*C*) The fluorescent colors of actinotrichia were changed by the photo-conversion at two regions of the dorsal side. On day 7 after photo-conversion, the positions of the labeled actinotrichia shifted to the more dorsal side, and the region of red fluorescence was compressed along the distal-proximal axis. Yellow dotted lines indicate the midline in the larval bodies. White dotted lines indicate the outline of notochords. Scale Bars, 50 μm.

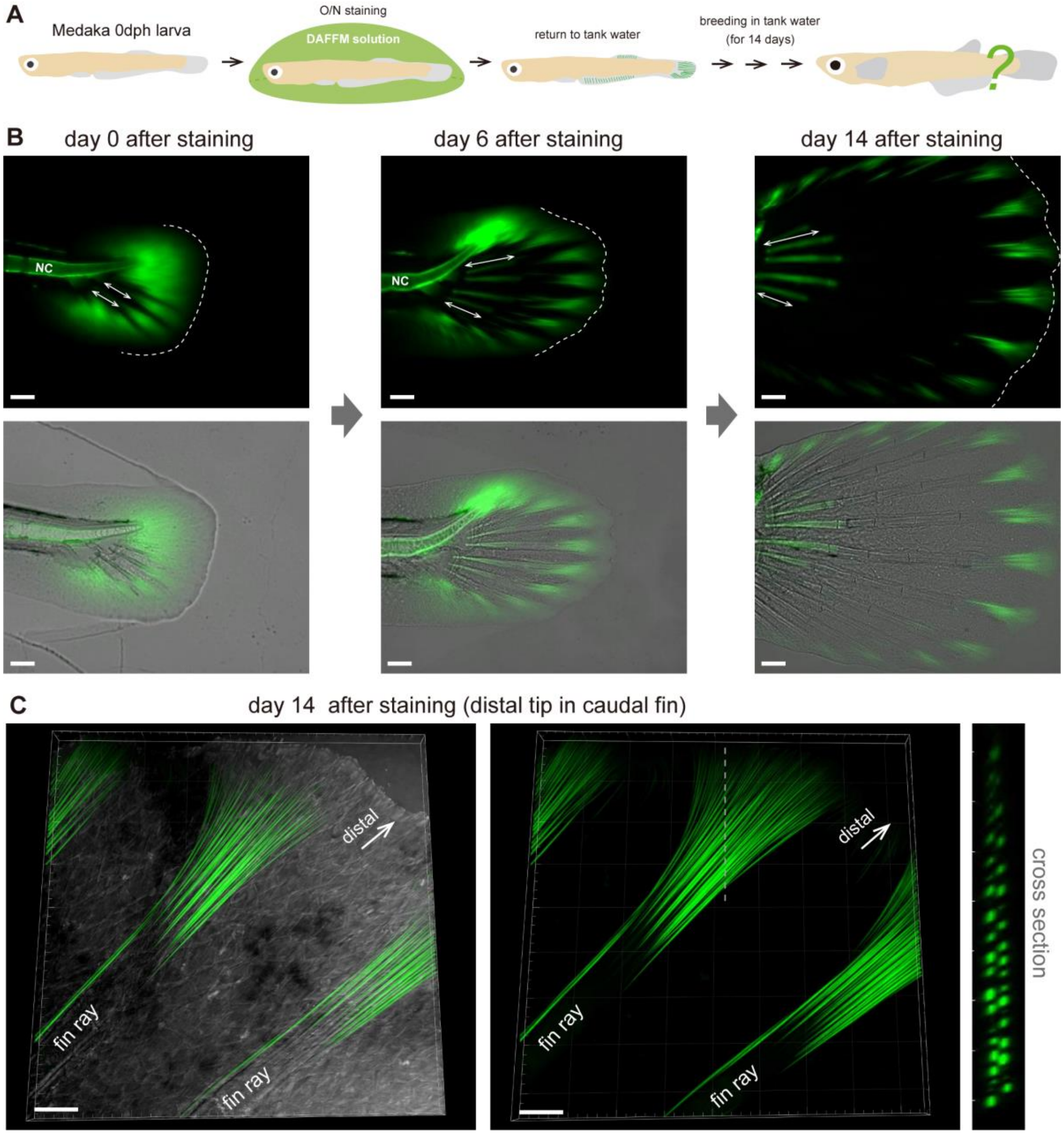

Fig. S8. Dynamic movement of actinotrichia in the direction of distal fin tip during fin growth of medaka. (*A*) Workflow for the pulse-chase observation of actinotrichia in medaka fins by DAFFM staining after hatching. Living medaka larvae were incubated overnight in DAFFM solution and then returned to tank water. After the staining, the distribution pattern of actinotrichia was observed over time during the 14 days of breeding. (*B*) The fluorescence images of the actinotrichia in the caudal fins at day 0, day 6 and day 14 after DAFFM staining. White dotted lines indicate the distal margins of caudal fins. Double arrows indicate the fluorescent signals in fin rays formed during 0dph (day post hatch) to 1dph. NC; notochord. (*C*) Confocal images of the fluorescently labeled actinotrichia in the caudal fin (day 14 after DAFFM staining). A cross-sectional view is shown in the right panel. Scale bars, 100 µm (*B*) and 50 µm (*C*).

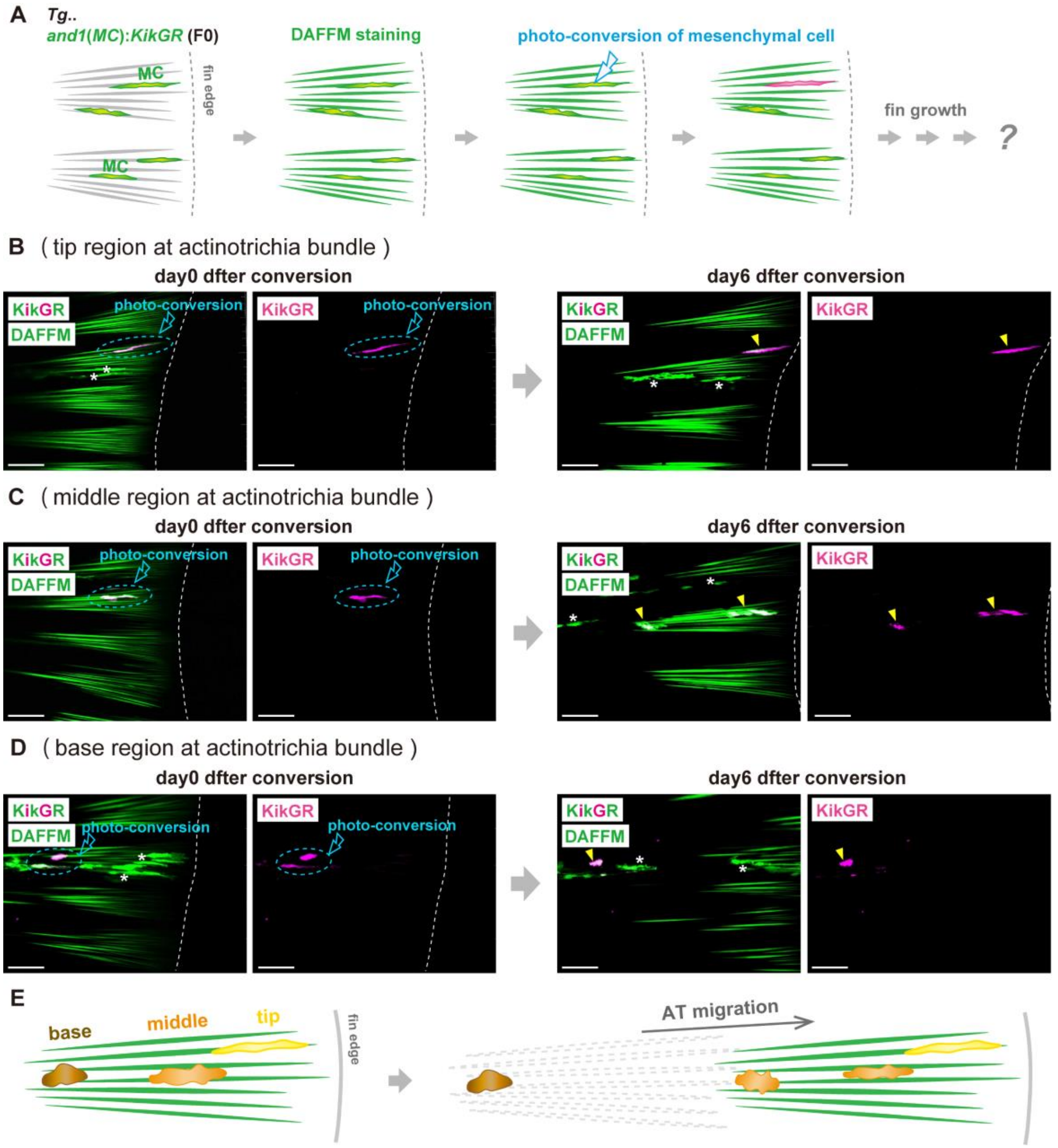

Fig. S9. Migration patterns of the mesenchymal cells during actinotrichia migration towards distal fin tip. (*A*) Workflow for the pulse-chase observation of mesenchymal cells and actinotrichia using DAFFM staining and photo-conversion. After DAFFM staining, the fluorescence color of the mesenchymal cells in F0 Tg zebrafish which express KikGR in fin mesenchymal cells were changed by UV stimulation. MC; mesenchymal cell. (*B*) The fluorescent color of the mesenchymal cell was changed by the photo-conversion at very tip region of the actinotrichia bundle. On day 6 after photo-conversion, the position of the labeled mesenchymal cell shifted towards distal fin tip together with actinotrichia. (*C*) The fluorescent color of the mesenchymal cell was changed by the photo-conversion at middle region of the actinotrichia bundle. On day 6 after photo-conversion, the labeled mesenchymal cell split into two cells, and the one of them positioned at base region of actinotrichia bundle. (*D*) The fluorescent color of the mesenchymal cell was changed by the photo-conversion at base region of the actinotrichia bundle. On day 6 after photo-conversion, the labeled mesenchymal cell retained their original positioning. White dotted lines indicate the distal fin margin. Yellow arrowheads indicate red KikGR fluorescence in

mesenchymal cells that were photo-converted 6 days ago. White asterisks indicate green KikGR fluorescence in mesenchymal cells that were not photo-converted. Scale Bars, 50 μm. (*E*) Schematic illustration of the migration patterns of the fin mesenchymal cells during actinotrichia (AT) migration towards distal tip. Fin mesenchymal cells exhibit different dynamics in each region.

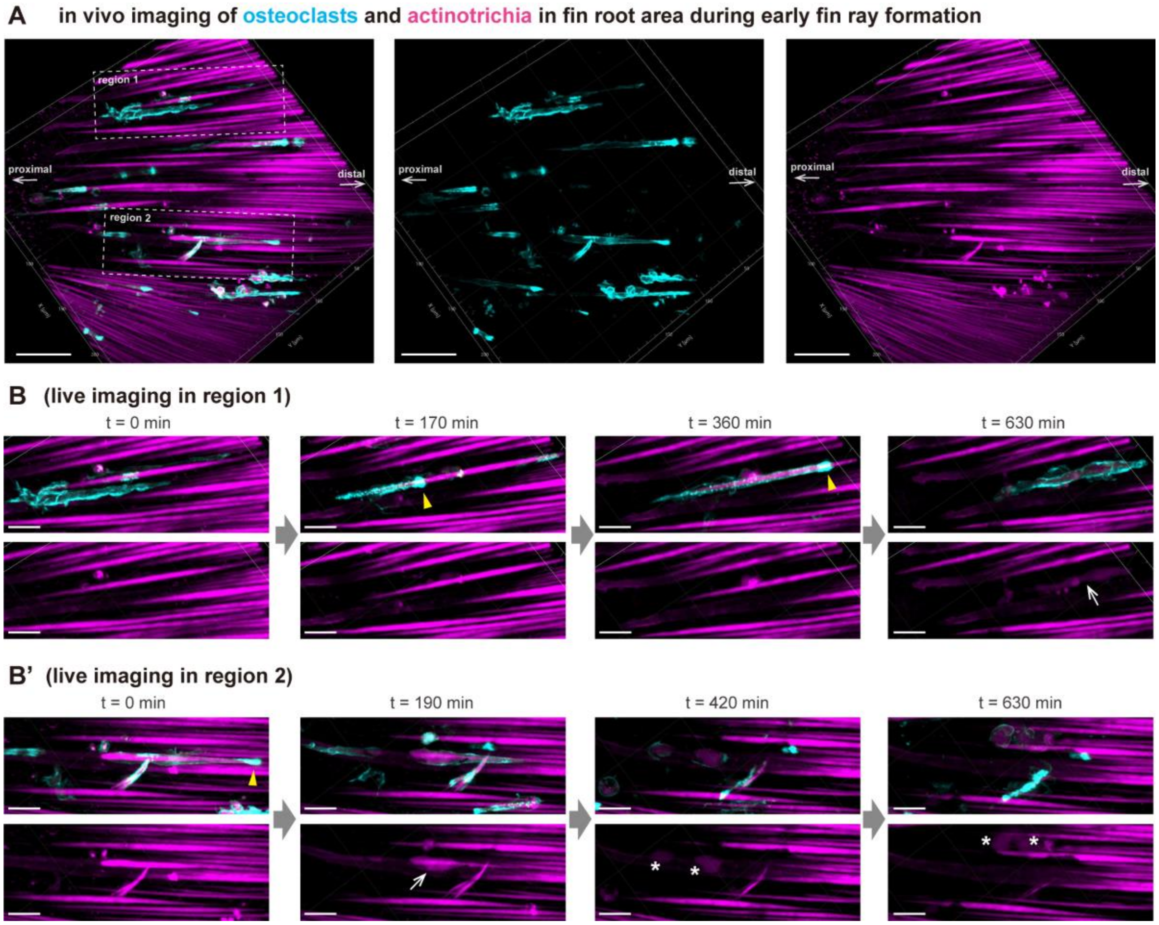

Fig. S10. Dynamics of the actinotrichia degradation in fin root area during early fin ray formation. Tg zebrafish larvae at 11dpf which express *lifeact-gfp* under the *TRAP* promoter were incubated overnight in DAR4M solution, and osteoclasts and the fluorescently labelled actinotrichia were imaged at the same position every 10 min by a confocal microscope. (*A*) Caudal fin root area observed in time-lapse analysis (x; 280 μm, y; 280 μm, z; 24 μm). Each osteoclast (cyan) started to hold a single actinotrichia (magenta) and elongate along the longitudinal axis of actinotrichia. (*B*) Images captured at different time points by the time-lapse analysis of region 1 in (*A*). *TRAP*-expressing osteoclast gradually elongated along the longitudinal axis of actinotrichia, wrapping around a single actinotrichia by accumulating actin (yellow arrowheads). After 630 min of imaging, actinotrichia surrounded by osteoclasts had undergone degradation and changed to an irregular shape (white arrow). (*B'*) Images captured at different time points by the time-lapse analysis of region 2 in (*A*). Initially, *TRAP*-expressing osteoclasts strongly accumulated actin at the distal end of a single actinotrichia (yellow arrowheads) and wrapped the actinotrichia structure. After 190 min, actinotrichia that were wrapped in osteoclasts became irregular in shape (white arrow). Subsequently, after 230 min, this irregularly shaped actinotrichia split into two fragments (white asterisks), and then, during the next 210 min, these two fragments were dynamically moving while being wrapped by osteoclasts. Scale bars, 50 μm (*A*) and 20 μm (*B*).

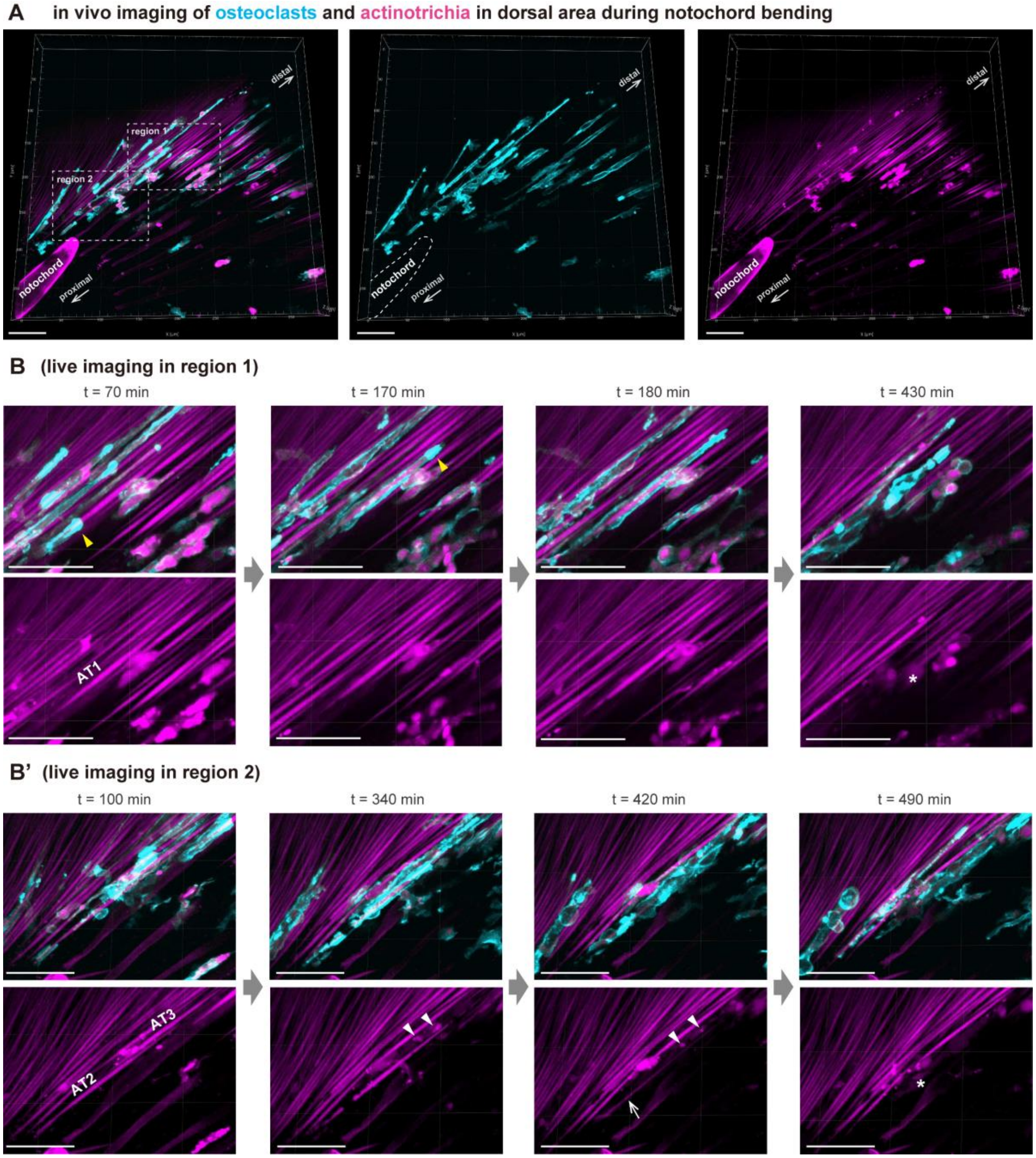

Fig. 11. Dynamics of the actinotrichia degradation in dorsal area during notochord bending. Tg zebrafish larvae at 14dpf which express *lifeact-gfp* under the *TRAP* promoter were incubated overnight in DAR4M solution, and osteoclasts and the fluorescently labelled actinotrichia were imaged at the same position every 10 min by a confocal microscope. (*A*) Dorsal area of the caudal fin observed in time-lapse analysis (x; 380 μm, y; 380 μm, z; 36 μm). A large number of osteoclasts (cyan) wrapped around the actinotrichia (magenta) while elongating. Many irregularly shaped actinotrichia were widely distributed. (*B*) Images captured at different time points by the time-lapse analysis of region 1 in (*A*). *TRAP*-expressing osteoclast started to hold one actinotrichia (AT1) by accumulating actin (yellow arrowheads) (t = 70 min). The cell gradually elongated along the longitudinal axis of AT1 while accumulating actin on its distal end (yellow arrowheads) (t = 170 min), and after further elongation, it completely wrapped the AT1 structure (t = 180 min). After that, it gradually regressed to the proximal side, and AT1 began to be degraded

and changed into an amorphous shape (white asterisk) (t = 430 min). (*B'*) Images captured at different time points by the time-lapse analysis of region 2 in (*A*). Two actinotrichia (AT2 and AT3) were wrapped by the osteoclasts with elongated morphology (t = 100 min). AT3 began to be degraded and divided into two small fragments (white arrowheads) (t = 340 min). Subsequently, AT2 began to change into thin and wavy shape (white arrow) (t = 420 min). AT2 continued to change its shape and became a small amorphous structure (white asterisk) (t = 490 min). Scale bars, 50 μm.

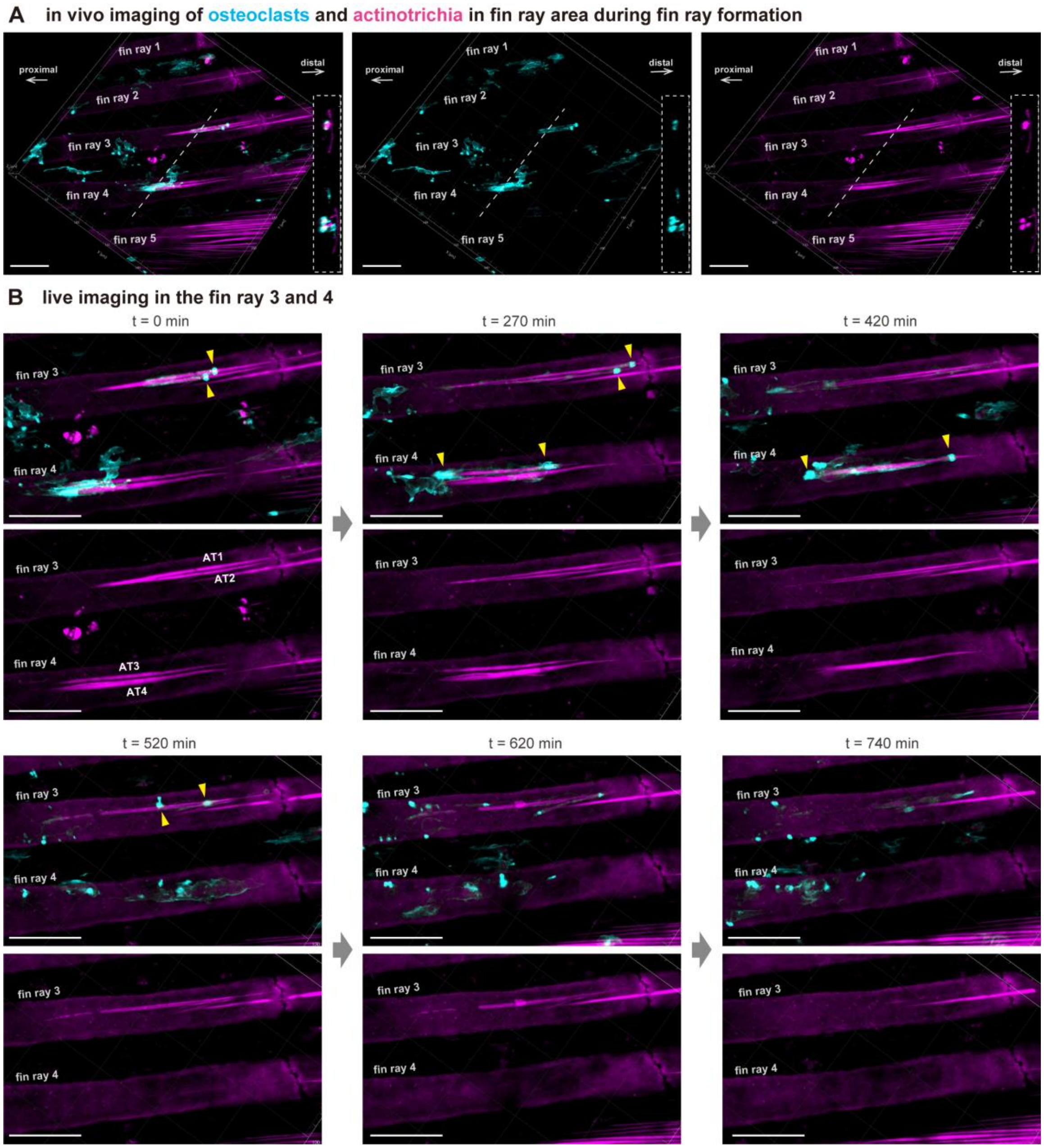

Fig. S12. Dynamics of the actinotrichia degradation in fin ray area during fin ray formation. Tg zebrafish larvae at 24dpf which express *lifeact-gfp* under the *TRAP* promoter were incubated overnight in DAR4M solution, and osteoclasts and the fluorescently labelled actinotrichia were imaged at the same position every 10 min by a confocal microscope. (*A*) Fin ray area of the caudal fin observed in time-lapse analysis (x; 300 μm, y; 300 μm, z; 27 μm). The right inset images in each fluorescence image show the cross-sectional views at the white dotted lines. Actinotrichia (magenta) distributed just below fin rays were surrounded with osteoclasts (cyan). (*B*) Images capturing at different time points by the time-lapse analysis of the actinotrichia in fin ray 3 and fin ray 4 (*A*). In fin ray 3, *TRAP*-expressing osteoclast hold two actinotrichia (AT1 and AT2) distributed just below fin ray 3 while accumulating actin at the distal side (Yellow arrowheads) (t = 0 min). During 270 min of imaging, the osteoclast moving actin-rich structures to more distal side. After the next 250 min, these actin-rich structures returned to the proximal side (t = 520 min), and during the next 220 min, AT1 and AT2 were degraded (t = 740 min). In fin ray 4, *TRAP*-expressing osteoclast started to interact with two actinotrichia (AT3 and AT4) (t =0 min).

During 270 min of imaging, the osteoclast gradually elongated along the longitudinal axis of AT3 while accumulating actin-rich structures at the proximal and the distal side (Yellow arrowheads). Subsequently, during the next 150 min, AT3 was degraded and completely disappeared, and then this osteoclast started to hold AT4 by accumulating actin-rich structures at proximal and the distal side (Yellow arrowheads) (t = 420 min). During the next 320 min, the cell lost actin accumulation, and the thin, elongated cell morphology gradually changed to a sac-like shape, and finally AT4 was degraded and disappeared (t = 740 min). Scale bars, 50 μm.

in vivo imaging of osteoclasts and actinotrichia in actinotrichia bundle during fin ray formation

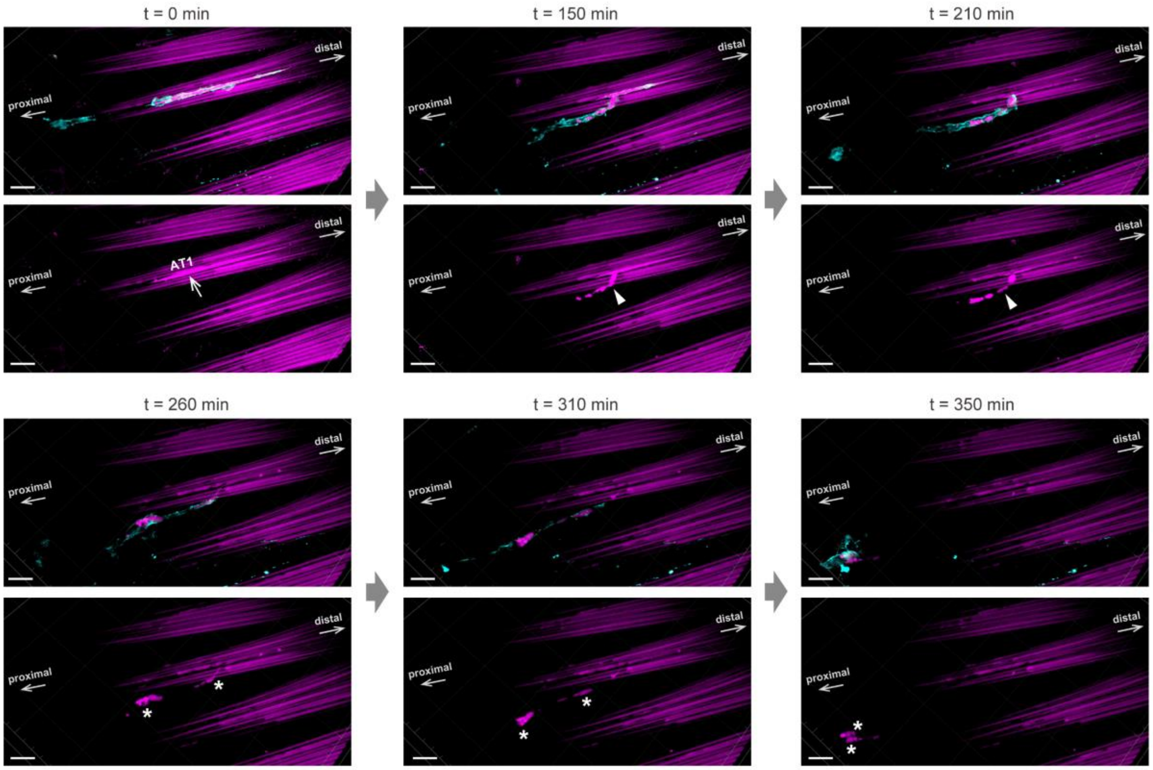

Fig. S13. Dynamics of the actinotrichia degradation in actinotrichia bundle during fin ray formation. Tg zebrafish larvae at 22dpf which express *lifeact-gfp* under the *TRAP* promoter were incubated overnight in DAR4M solution, and osteoclasts (cyan) and the fluorescently labelled actinotrichia (magenta) were imaged at the same position every 10 min by a confocal microscope (x; 290 μm, y; 290 μm, z; 28 μm). Initially, *TRAP*-expressing osteoclast elongated along the longitudinal axis of the single actinotrichia (AT1, white arrow) and wrapped the actinotrichia structure (t = 0 min). During 150 min of imaging, AT1 that was wrapped in the osteoclast became irregular in shape at the proximal side (white arrowhead) and started to move toward the proximal direction. After the next 60 min, AT1 continued to change the shape and started to become discontinuous (t = 210 min). AT1 was subsequently divided into two irregularly shaped fragments (white asterisks) wrapped by the osteoclast (t = 260 min), their positions shifted toward a more proximal direction along with the dynamic morphological changes of the osteoclast (t = 310min). Finally, two small and irregularly shaped fragments came close to each other, wrapped by the osteoclast that contracted in the distal-proximal direction (t = 350 min). Scale bars, 20 μm.

Movie S1. Cross-sectional image series of the confocal images showing the three-dimensional orientation of actinotrichia. Actinotrichia in the larval fins at 5dpf were labeled with DAFFM. The fluorescently labelled actinotrichia (green) and the cell nuclei (blue) stained with hoechst in the caudal fin were imaged by a confocal microscope (x; 142 μm, y; 142 μm, z; 15 μm).

Movie S2. 3D rotation movie of the actinotrichia stained with DAFFM and DAR4M shown in Fig. 1G to G'. The fluorescently labelled actinotrichia (DAFFM: green, DAR4M: magenta) in the caudal fin were imaged by a confocal microscope (x; 152 μm, y; 152 μm, z; 14 μm).

Movie S3. A time-lapse analysis of the actinotrichia showing morphological changes in the fins of a live fish. Actinotrichia in the larval fins at 12dpf were labeled with DAFFM. The fluorescently labelled actinotrichia (green) in the caudal fin were scanned at the same position every 10 min by a confocal microscope (x; 425 μm, y; 425 μm, z; 30 μm).

Movie S4. Growth dynamics of actinotrichia revealed by pulse chase observation using two different fluorescent probes. Actinotrichia present before 40 days are indicated by DAFFM (green), and actinotrichia grown for 40 days are indicated by DAR4M (magenta). The fluorescence of actinotrichia in the dorsal area of the caudal fin were imaged by a confocal microscope (x; 425 μm, y; 425 μm, z; 15 μm).

Movie S5. 3D rotation movie of the physical interaction between actinotrichia and osteoclasts. Actinotrichia in the larval fins at 22dpf were labeled with DAR4M. The fluorescently labelled actinotrichia (magenta) and the osteoclasts (cyan) expressing *lifeact-gfp* under the *TRAP* promoter in the caudal fin were imaged by a confocal microscope (x; 425 μm, y; 425 μm, z; 16 μm).

Movie S6. A timelapse movie of the actinotrichia in the degradation process shown in Fig. 7D. Actinotrichia in the larval fins at 12dpf were labeled with DAR4M. The fluorescently labelled actinotrichia (magenta) and the osteoclasts (cyan) expressing *lifeact-gfp* under the *TRAP* promoter were scanned at the same position every 10 min by a confocal microscope (x; 283 μm, y; 283 μm, z; 23 μm).

Movie S7. A timelapse movie of the actinotrichia in the degradation process shown in fig. S7. Actinotrichia in the larval fins at 11dpf were labeled with DAR4M. The fluorescently labelled actinotrichia (magenta) and the osteoclasts (cyan) expressing *lifeact-gfp* under the *TRAP* promoter were scanned at the same position every 10 min by a confocal microscope (x; 280 μm, y; 280 μm, z; 24 μm).

Movie S8. A timelapse movie of the actinotrichia in the degradation process shown in fig. S8. Actinotrichia in the larval fins at 14dpf were labeled with DAR4M. The fluorescently labelled actinotrichia (magenta) and the osteoclasts (cyan) expressing *lifeact-gfp* under the *TRAP* promoter were scanned at the same position every 10 min by a confocal microscope. (x; 380 μm, y; 380 μm, z; 36 μm).

Movie S9. A timelapse movie of the actinotrichia in the degradation process shown in fig. S9. Actinotrichia in the larval fins at 24dpf were labeled with DAR4M. The fluorescently labelled actinotrichia (magenta) and the osteoclasts (cyan) expressing *lifeact-gfp* under the *TRAP* promoter were scanned at the same position every 10 min by a confocal microscope. (x; 300 μm, y; 300 μm, z; 27 μm).

Movie S10. A timelapse movie of the actinotrichia in the degradation process shown in fig. S10. Actinotrichia in the larval fins at 22dpf were labeled with DAR4M. The fluorescently labelled actinotrichia (magenta) and the osteoclasts (cyan) expressing *lifeact-gfp* under the *TRAP* promoter were scanned at the same position every 10 min by a confocal microscope (x; 290 μm, y; 290 μm, z; 28 μm).

SI Materials and Methods

Animal strains and maintenance. Zebrafish were maintained under the standard laboratory conditions and treated as previously described (6). AB strains were used as wild type zebrafish. The following transgenic (Tg) lines were used in this study: *Tg* (*col2a1a: H2B-mRFP*), *Tg* (*and1 1.4k: And1^{480bp}-KikGR*) (3), *Tg (5×and1(MC): KikGR)*, *Tg (5×and1(MC): lifeact-mCherry)* (3), *Tg* (*osx: lifeact-mCherry*) (7), *Tg* (*3×osx: lifeact-YFP*) (8), *Tg* (*TRAP: lifeact-GFP*). 1.7 kb upstream regulatory region of the zebrafish *col2a1a* gene was used as a *col2a1a* promoter for the generation of *pTol2-col2a1a: H2B-mRFP*. 6 kb upstream regulatory region of the zebrafish *TRAP* (*acp5a*) gene was used as a *TRAP* promoter for the generation of *pTol2-TRAP: lifeact-GFP*. *pTol2-5xand1(MC): KikGR* was made as previously described (3, 9). These Tg lines were generated by injecting the tol2 plasmid with Tol2 transposase (10). Japanese medaka (Oryzias latipes) were bred and maintained under the conditions with 14 and 10 h of light (8:30-22:30) and dark cycles at 25 °C. They were fed commercial powder food 2 times a day. All zebrafish and medaka experiments were approved by the animal care and use at Osaka University.

Microscopy for the fluorescent imaging. Before observation of live samples, zebrafish were anesthetized with tricaine (MS-222) at an optimal concentration according to each body size. Anesthetized zebrafish were placed on a 35mm glass bottom dish (Iwaki, 3910-035) and used for imaging. The images were obtained using a fluorescence microscope, BZ-X710 (Keyence) with 10× NA 0.45 Plan Apo and 20× NA 0.45 Plan Fluor objective (Nikon) and a confocal microscope, LSM 780 (Carl Zeiss) with 20× NA 0.8 Plan Apo (Carl Zeiss) and 40× NA 1.4 Oil Plan Apo objective (Carl Zeiss) and a two-photon microscope, A1R MP+/Ti2-E (Nikon) with Apo LWD 20× WI (Nikon). 890 nm laser excitation and a 440 nm SP emission filter was used for SHG imaging of actinotrichia. ZEN (Carl Zeiss) and Imaris (Bitplane) were used as image software for z projections. Live cell imaging was performed using a LSM780 (Carl Zeiss) with 20× NA 0.8 Plan Apo (Carl Zeiss) (for Fig. 7, fig. S7, fig. S8 and fig. S9) and a STELLARIS8 (Leica) with HC PL Apo CS2 20×/0.75 (Leica) (for fig. S10). The time-lapse image data were processed into videos using Imaris (Bitplane).

DAFFM/DAR4M treatment for actinotrichia visualization. DAF-FM DA (Goryo Chemical, SK1004-01) and DAR-4M AM (Goryo Chemical, SK1006-01) were used for zebrafish whole mount staining to visualize actinotrichia. DAF-FM DA and DAR-4M AM were diluted with fresh breeding water and adjusted to concentrations of 5 μM and 10 μM, respectively, and used for staining. Dilute solutions of DAFFM and DAR4M should be freshly prepared and adjusted each time before use for staining. Living zebrafish were bathed in each staining solution in dark conditions and treated for 1-12 hr at room temperature. 12-well or 6-well microplates (Iwaki, flat bottom) were used for DAFFM/DAR4M staining depending on fish size. After staining, zebrafish were immediately returned to fresh breeding water and then grown under room light irradiation without shading until observation. For the imaging analysis using a confocal microscope, the fluorescent signals of DAFFM and DAR4M were detected by a GaAsP detector with 488 nm and 561 nm lasers, respectively.

NOS inhibition and NO removal. To investigate the involvement of cell-produced NO in the process of actinotrichia luminescence by DAFFM, actinotrichia were first isolated from zebrafish larval fins according to the method reported in previous studies (3). Actinotrichia isolated from fin tissues and transferred in the culture dish were then treated under three different conditions: DMSO (0.1% in PBS) as control, N^G-nitro-L-arginine methyl ester hydrochloride (L-NAME, Dojindo, N412) (1mM in PBS) known as an inhibitor of NOS, and 2-(4-Carboxyphenyl)-4,4,5,5-tetramethylimidazoline-1-oxyl-3-oxide (C-PTIO, Dojindo, C348) (500μM in PBS) known as a NO scavenger. In the control and L-NAME treatment experiment, after 24 hr of the treatment, actinotrichia were incubated for 12 hr at room temperature in DAFFM solution (5 μM) diluted with the corresponding drug solution (0.1% DMSO and 1mM L-NAME). In C-PTIO treatment experiment, after 30 min of the treatment, actinotrichia were incubated in DAFFM solution (5 μM) diluted with C-PTIO solution (500μM) for 12 hr at room temperature. After DAFFM staining, fluorescent images of actinotrichia under each condition were captured using a confocal

microscope and the fluorescence intensity values were measured by FIJI image analysis software.

Photo-conversion of actinotrichia in larval fins. Larval zebrafish of *Tg (and1 1.4k: And1^{480bp}-KikGR)* were used for photo-conversion of actinotrichia. Before the photo-conversion of actinotrichia, Tg fish were anesthetized with tricaine (MS-222) at an optimal concentration. Anesthetized fish were placed on a 35mm glass bottom dish (Iwaki, 3910-035) and actinotrichia in their fins were region-specifically stimulated with UV light (405nm) until their fluorescent color fully changed using LSM780 microscopy (Carl Zeiss) with 20× NA 0.8 Plan Apo (Carl Zeiss). After the photo-conversion, Tg fish were immediately returned to fresh breeding water and then grown in the dark condition until next observation.

Photo-conversion of mesenchymal cells in larval fins. Mosaic F0 zebrafish of *Tg (5xand1(MC): KikGR)* bred in dark conditions were used for photo-conversion of mesenchymal cells distributed in fin tip region. Before the photo-conversion of mesenchymal cells, F0 Tg fish were treated with DAF-FM DA solution (5μM) in dark conditions at room temperature for 12 hours. Subsequently, F0 Tg fish were briefly washed with fresh breeding water and anesthetized with tricaine (MS-222) at an optimal concentration. Then, anesthetized fish were placed on a 35mm glass bottom dish (Iwaki, 3910-035) and single mesenchymal cell whish express KikGR at high level was specifically stimulated with UV light (405nm) until their fluorescent color fully changed using LSM780 microscopy (Carl Zeiss) with 20× NA 0.8 Plan Apo (Carl Zeiss). After the photo-conversion, F0 Tg fish were immediately returned to fresh breeding water and then grown in dark conditions until next observation 6 days later.

Evaluation of the effect of DAFFM treatment on the growth of zebrafish. Larval zebrafish at 6dpf were treated with DAF-FM DA solution (5μm) in dark conditions at room temperature for 12 hours. As a control for the DAFFM treatment group, larval fish at the same stage were treated with DMSO (0.1%). 50 larval fish in both groups were used for each treatment. After treatment, they were returned to fresh breeding water and grown for one month under room light irradiation. The survival rate of each group was counted after one month of growth. The same test was performed a total of three times. The fish in the control and DAFFM-treated groups were stained with Hoechst (Dojindo) and the fluorescence of the nuclei of the cells comprising caudal fins was taken using a fluorescence microscope, BZ-X710 (Keyence) with 10× NA 0.45 Plan Apo. Each fish was stained with Alizarin Red (Wako) and the calcified fin bones were fluorescently visualized. The proximal end of the fin bone was defined as the root of the fin, and the areas were calculated. The number of fin bones and the fluorescence intensity of Alizarin Red were also measured.

Fin regeneration assay. Old mature zebrafish (1.5-year-old) were used for the fin regeneration experiment. The actinotrichia of old mature fish were fluorescently labeled by treatment with DAF-FM DA solution (5μM) in dark conditions at room temperature for 12 hours, and after a brief subsequent washing, fluorescent images of the tip region of caudal fins at ventral side were immediately taken using a fluorescence microscope, BZ-X710 (Keyence) with 10× NA 0.45 Plan Apo. Then, the fish were anesthetized with tricaine (MS-222) at an optimal concentration, and the tip region of three fin bones (V2, V3 and V4) at ventral side of caudal fins were amputated with surgical knives. After the fin amputation, the fish were immediately returned to fresh breeding water and maintained in tanks of circulation system for 40 days. Finally, the same areas of the caudal fin tip were observed again, and fluorescent images of the regenerated fins were obtained.

BrdU incorporation and immunostaining. Living zebrafish larvae were incubated with 5mM of 5-Bromo-2′-deoxyuridine (BrdU, Abcam, ab142567) diluted in fresh breeding water with 0.1% DMSO for 1 hr at room temperature. After incubation, larvae were fixed with 4% paraformaldehyde (PFA) in PBS O/N at 4°C. After the fixation, the samples were washed with PBS-0.2% tween 20 (PBST) and immersed in methanol. Subsequently, they were transferred to acetone and incubated for 20 min at -30°C. After cold treatment, they were washed with PBST and treated with 2N HCl for 20 min at room temperature for permeabilization. They were then

washed with PBST and incubated in blocking solution (5% goat serum in PBST) for 1 hr at room temperature. After the blocking, they were incubated with mouse monoclonal anti-BrdU antibody (Invitrogen; 1:200) solution (5% goat serum in PBST) O/N at 4°C. Next day, they were washed with PBST and incubated with secondary antibody and Hoechst (Dojindo; 1:200) solution (5% goat serum in PBST) for 2 hr at room temperature. After subsequent washing with PBS, they were used for the confocal microscopic imaging. Alexa Fluor 555 goat anti-mouse IgG antibody (Invitrogen; 1:200) was used as secondary antibody.

Staining of actin cytoskeleton in larval fins. The larval caudal fins were fixed with 4% PFA in PBS O/N at 4°C. After the fixation, the fin samples were washed with PBS and incubated with Phalloidin-iFluor 594 conjugated (AAT Bioquest; 1:300) in PBS O/N at 4°C. Subsequently, the samples were wash with PBS and used for confocal microscopic imaging.

TRAP staining. Osteoclast activities were detected by tartrate-resistant acid phosphatase (TRAP) staining kit (Cosmo Bio, AK04F). Wild type zebrafish larvae were fixed with 4% PFA overnight at 4℃ after anesthesia with MMS. After the fixation, samples were washed with PBS followed by distilled water each three times. Chromogenic substrate was dissolved in tartrate-containing buffer for the preparation of staining solution, and samples were incubated in staining solution for 1-2 hours at 37℃. Following incubation at 37℃, samples were washed with distilled water to stop the reaction, and bright field images of the fins were acquired with a BZ-X710 (Keyence) with 10× NA 0.45 Plan Apo and 20× NA 0.45 Plan Fluor objective (Nikon).

Live imaging of actinotrichia and osteoclasts. Living zebrafish of osteoclast reporter strain *Tg* (*TRAP: lifeact-GFP*) were treated with DAR4M solution at room temperature overnight and actinotrichia in their fins were labeled with red fluorescence. After DAR4M staining, Tg fish were anesthetized with MMS and their fins were amputated near the base under anesthesia condition. The amputated fin samples were embedded in stock solution of Matrigel (Corning, 356237) placed on a 35 mm glass bottom dish (Iwaki, 3910-035). The Matrigel samples were immediately transferred to a 37°C incubator and incubated for 30 min to harden. L-15 medium (Thermo Fisher Scientific, 21083-027) containing 10% FBS (Thermo Fisher Scientific, 16170-078) was added to the culture dish and the Matrigel samples were soaked in the culture medium. Time-lapse analyses for the observation of interaction between actinotrichia and osteoclasts were performed at 28℃ using a LSM 780 (Carl Zeiss) with 20× NA 0.8 Plan Apo (Carl Zeiss) and a STELLARIS8 (Leica) with HC PL Apo CS2 20×/0.75 (Leica), and the areas from z = 20 to 40 μm were scanned at 10 min intervals with a frame size of 1024 × 1024.

Statistical analysis. Statistical analysis was performed using GraphPad Prism software versions 9.5.1 (731). All data are expressed as means ± SD. Two-tailed Student's unpaired t test was used to determine the significance of the difference between means of two groups. ANOVA test with Tukey's multiple comparisons test was applied for the analysis of the statistical significance of differences among more than two groups. P-values are summarized as **p < 0.01, ***p < 0.001 and ****p < 0.0001 and indicated in the figure legends.

SI Appendix References

1. S. Lepiller, *et al.*, Imaging of nitric oxide in a living vertebrate using a diaminofluorescein probe. *Free Radical Biology and Medicine* **43**, 619–627 (2007).

2. J. Renn, B. Pruvot, M. Muller, Detection of nitric oxide by diaminofluorescein visualizes the skeleton in living zebrafish. *J. Appl. Ichthyol.* **30**, 701–706 (2014).

3. J. Kuroda, T. Itabashi, A. H. Iwane, T. Aramaki, S. Kondo, The Physical Role of Mesenchymal Cells Driven by the Actin Cytoskeleton Is Essential for the Orientation of Collagen Fibrils in Zebrafish Fins. *Front. Cell Dev. Biol.* **8**, 580520 (2020).

4. Pk. Böckelmann, Ij. Bechara, Influence of indomethacin on the regenerative process of the tail fin of teleost: morphometric and ultrastructural analysis. *Braz. J. Biol.* **70**, 889–897 (2010).

5. M. B. Hawkins, K. Henke, M. P. Harris, Latent developmental potential to form limb-like skeletal structures in zebrafish. *Cell* **184**, 899-911.e13 (2021).

6. ZFIN Publication: Westerfield, 1995. Available at: https://zfin.org/ZDB-PUB-970327-24 [Accessed 24 November 2023].

7. J. Kobayashi-Sun, *et al.*, Uptake of osteoblast-derived extracellular vesicles promotes the differentiation of osteoclasts in the zebrafish scale. *Commun Biol* **3**, 190 (2020).

8. H. Hino, S. Kondo, J. Kuroda, In vivo imaging of bone collagen dynamics in zebrafish. *Bone Rep* **20**, 101748 (2024).

9. T. Aramaki, S. Kondo, Independent size regulation of bones and appendages in zebrafish. [Preprint] (2024). Available at: https://www.biorxiv.org/content/10.1101/2024.03.27.587111v1 [Accessed 10 May 2024].

10. A. Urasaki, G. Morvan, K. Kawakami, Functional Dissection of the *Tol2* Transposable Element Identified the Minimal *cis* -Sequence and a Highly Repetitive Sequence in the Subterminal Region Essential for Transposition. *Genetics* **174**, 639–649 (2006).
